# The Basolateral Amygdala Is Essential for Rapid Escape: A Human and Rodent Study

**DOI:** 10.1016/j.cell.2018.09.028

**Published:** 2018-10-18

**Authors:** David Terburg, Diego Scheggia, Rodrigo Triana del Rio, Floris Klumpers, Alexandru Cristian Ciobanu, Barak Morgan, Estrella R. Montoya, Peter A. Bos, Gion Giobellina, Erwin H. van den Burg, Beatrice de Gelder, Dan J. Stein, Ron Stoop, Jack van Honk

**Affiliations:** 1 Department of Psychology, Utrecht University, Utrecht, the Netherlands; 2 Department of Psychiatry and Mental Health, University of Cape Town, Cape Town, South Africa; 3 Center for Psychiatric Neuroscience, Lausanne University and University Hospital Center, Lausanne, Switzerland; 4 Donders Institute for Brain, Cognition and Behaviour, Radboud University Nijmegen Medical Centre, Nijmegen, the Netherlands; 5 Global Risk Governance Program, Institute for Safety Governance and Criminology, Law Faculty, University of Cape Town, Cape Town, South Africa; 6 Department of Psychology and Neuroscience, Maastricht University, Maastricht, the Netherlands; 7 MRC Unit on Risk and Resilience in Mental Disorders, University of Cape Town, Cape Town, South Africa; 8 Institute of Infectious Disease and Molecular Medicine, University of Cape Town, Cape Town, South Africa

**Keywords:** basolateral amygdala, central amygdala, Urbach Wiethe, fear, freezing, startle reflex, threat, escape, DREADD, oxytocin

## Abstract

Rodent research delineates how the basolateral amygdala (BLA) and central amygdala (CeA) control defensive behaviors, but translation of these findings to humans is needed. Here, we compare humans with natural-selective bilateral BLA lesions to rats with a chemogenetically silenced BLA. We find, across species, an essential role for the BLA in the selection of active escape over passive freezing during exposure to imminent yet escapable threat (T_imm_). In response to T_imm_, BLA-damaged humans showed increased startle potentiation and BLA-silenced rats demonstrated increased startle potentiation, freezing, and reduced escape behavior as compared to controls. Neuroimaging in humans suggested that the BLA reduces passive defensive responses by inhibiting the brainstem via the CeA. Indeed, T_imm_ conditioning potentiated BLA projections onto an inhibitory CeA pathway, and pharmacological activation of this pathway rescued deficient T_imm_ responses in BLA-silenced rats. Our data reveal how the BLA, via the CeA, adaptively regulates escape behavior from imminent threat and that this mechanism is evolutionary conserved across rodents and humans.

## Highlights

• Human bilateral BLA damage and silencing in rats results in maladaptive passive fear
• If active escape is feasible, the BLA prevents passive freezing responses via the CeA
• BLA action on an inhibitory CeA pathway permits the adaptive shift to active escape
• Activation of CeA neurons by oxytocin rescues deficient escape in BLA-silenced rats

## Graphical Abstract

**Figure:**
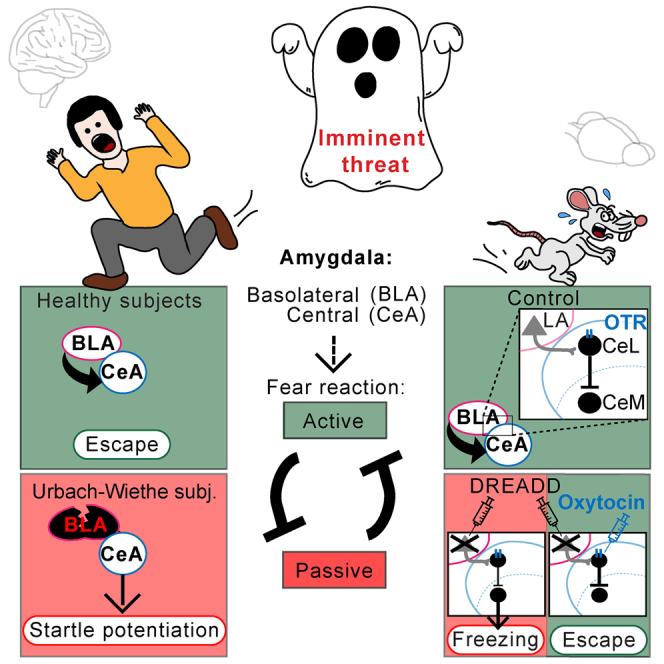

## Teaser

Under conditions of imminent threat, by activating an inhibitory central amygdala pathway, the rodent and human basolateral amygdala play a key role in adaptively selecting and executing active escape responses rather than passive freezing behaviors.

## Introduction

The amygdala is an almond-shaped group of nuclei in the temporal lobe that is crucial for defensive behaviors in all mammals, including humans (Davis and Whalen, 2001, Phelps and LeDoux, 2005). The nuclei of the amygdala are partly of striatal origin (central amygdala [CeA]) and partly of cortical origin (basolateral amygdala [BLA]), with the BLA in particular having undergone a large evolutionary expansion in humans compared to rodents (Janak and Tye, 2015). Cross-species translational research on the behavioral functions of this circuitry is thus essential. A seminal body of rodent research has delineated how amygdala circuitry controls defensive behaviors (Ciocchi et al., 2010, Fadok et al., 2017, Gozzi et al., 2010, Haubensak et al., 2010, Li et al., 2013, Tovote et al., 2016, Tye et al., 2011, Viviani et al., 2011), but cross-species translation is a challenge (Janak and Tye, 2015), because in human studies, evidence of causal mechanisms and sub-region specificity are lacking.

Recently, we identified a group of humans with focal, bilateral calcifications in the BLA; these patients can potentially provide such causal evidence. BLA damage in these individuals is caused by an extremely rare autosomal recessive disorder—Urbach-Wiethe disease (UWD) (Hamada et al., 2002)—which was introduced to South Africa with the arrival of Dutch-German settlers in 1652 and continued to spread locally because of the founder effect (Van Hougenhouck-Tulleken et al., 2004). Using structural and functional neuroimaging methods in affected individuals, we recently demonstrated that, in this group, there is a focal bilateral neurodegeneration that is restricted to within the BLA without affecting CeA (Terburg et al., 2012). This group of individuals could therefore contribute significantly to cross-species translation of functions of the different nuclei in the amygdala.

In rodents, the BLA is crucial for threat conditioning (Davis and Whalen, 2001). Although initial behavioral results in the South-African UWD population found that BLA pathology reduced acquisition of threat-potentiated startle (Klumpers et al., 2015), such pathology also increased vigilance for facial and bodily threat stimuli across multiple behavioral paradigms (de Gelder et al., 2014, Hortensius et al., 2016, Terburg et al., 2012). This hyper-vigilance for threat was also observed in response to non-consciously processed threat stimuli in a paradigm precluding cortical control (Terburg et al., 2012). This suggested that threat reactivity downstream of the BLA is potentiated by BLA damage (Liddell et al., 2005, van Honk et al., 2002, van Honk et al., 2005). In rodent models, there is also evidence that threat reactivity is increased after BLA inhibition (Macedo et al., 2006) and that anxious behaviors are increased by inhibition of BLA projections to the lateral CeA (CeL) (Tye et al., 2011). Recent evidence furthermore indicates that the CeL can act as a switch between passive—freezing—and active—escape—defensive behaviors, specifically due to inhibitory projections to the medial subdivision (CeM) and the brainstem (Ciocchi et al., 2010, Fadok et al., 2017, Gozzi et al., 2010, Haubensak et al., 2010, Tovote et al., 2016, Viviani et al., 2011). Given that the BLA-CeL pathway can activate these inhibitory projections to the CeM (Tye et al., 2011), we hypothesized that BLA input is necessary to induce the passive to active switch, that is the switch from freezing to active escape, in the CeA. Neuroimaging in humans indeed suggests that the BLA is involved in goal-directed active escape behavior (Mobbs et al., 2007), but direct and causal evidence for this model and its cross-species translation is currently unavailable.

We therefore set out to investigate BLA-damaged human subjects in parallel with rodents with the aim of translating across species and elucidating the role of the BLA and CeA in active compared to passive defensive behaviors. Under threat, mammals, including humans, can either respond passively with defensive reactions, such as freezing, or actively by goal-directed actions, such as escape (McNaughton and Corr, 2004, Mobbs et al., 2007, Montoya et al., 2015). The neural mechanism that selects when to freeze and when to escape is, however, not well understood (LeDoux et al., 2017). Accordingly, we developed cross-species paradigms that extend recent research on responses to inescapable threat (Fadok et al., 2017, Klumpers et al., 2015) to situations where behavior can lead to successful escape. Outcome measures included behavioral escape performance and freezing, acoustic startle reflexes, fMRI, and *ex vivo* cell recordings.

By comparing in both species individuals with and without a functional BLA, we identified a vital inhibitory control function of the BLA over passive defensive behaviors. This inhibitory control by the BLA was adaptively tuned, that is, selectively present in situations of imminent threat that requested rapid escape and not present in distant or inescapable threat conditions. Neuroimaging evidence in humans suggested that, to execute this inhibitory control function, the BLA acts on the CeA in order to influence the downstream brainstem and its initiation of passive defensive behavior. In rodents, we found that training by exposure to imminent escapable threat selectively upregulated BLA projections to a group of neurons in the CeL identifiable by their sensitivity to oxytocin. By increasing the oxytocin signaling in the CeA (Huber et al., 2005, Knobloch et al., 2012, Viviani et al., 2011), we rescued the responses to imminent escapable threat during downregulated function of the BLA. Together, these cross-species findings not only uncover a dynamically tuned regulation by the BLA of the CeA under conditions of imminent escapable threat but also show how this is mechanistically established by the BLA via activation of inhibitory projections from the CeL to CeM. Finally, this key role of the BLA in escape behavior is evolutionary conserved across humans and rodents.

## Results

### Calcifications Are Bilateral and Focal to the Human BLA

**Figure 1:**
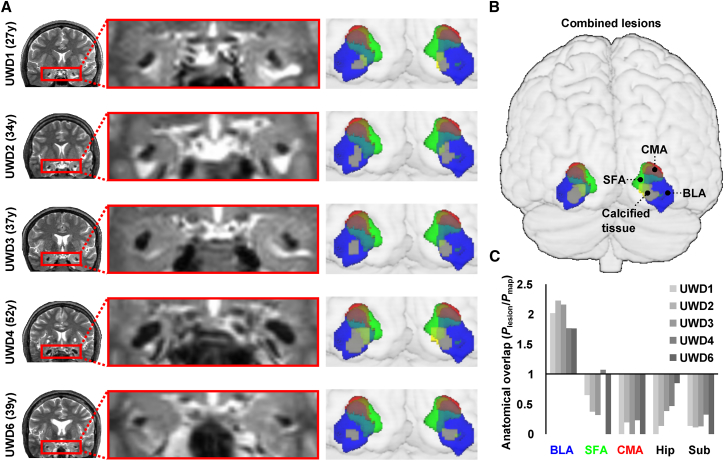
Calcifications Are Bilateral and Focal to the BLA (A) Coronal slices from each individual’s T2-weighted MRI scan, age at time of scanning, and in MNI-space-estimated lesion volumes plotted within the amygdala sub-regions (voxel-probability > 50%; see STAR Methods). (B) Combined lesions image showing all five lesion volumes. (C) Bilateral excess probability (*P*_excess_) values of the lesion volumes, whereby values >1 indicate a reliable match of volume and anatomical location. BLA, basolateral amygdala; CMA, central-medial amygdala; Hip, hippocampus; SFA, superficial amygdala; Sub, subiculum. See Video S1 for 3D renderings of lesion volumes. See also Figure S1.

We first obtained high-resolution T2-weighted MRIs of each of the five UWD subjects included in this study. Using an MRI probability-mapping method described by Eickhoff et al. (2005), we were able to quantify the overlap of each calcification with cytoarchitectonic structure-probability maps of the amygdala sub-regions developed by Amunts et al. (2005). In line with our previous findings in this group (Terburg et al., 2012), in each of the five UWD subjects, we found bilateral calcifications that were localized to the BLA without affecting the CeA (Figure 1; Video S1).

Video S1. 3D Renderings of Lesion Volumes, Related to Figure 1

### BLA Damage Leads to Increased Startle Potentiation to Imminent Escapable Threat

**Figure 2:**
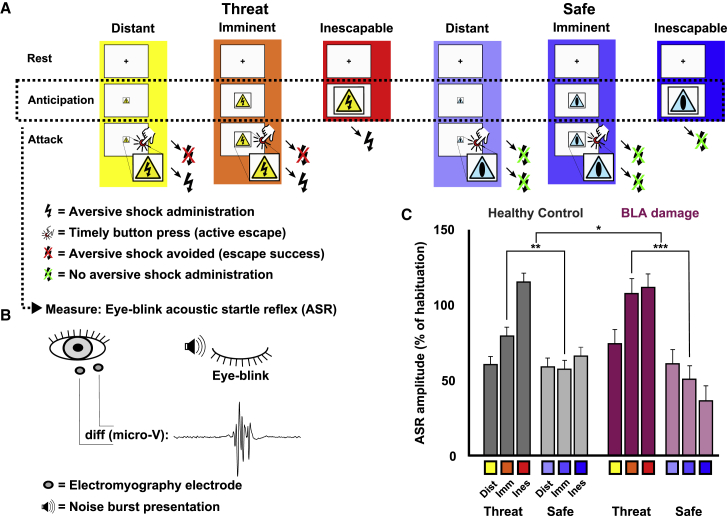
BLA Damage Leads to Over-potentiation of the ASR during Anticipation of Imminent yet Escapable Threat (A) Participants in the TET saw pictures that could “attack” by a rapid approach, during which only a sufficiently fast button press could provide escape. Escape failure resulted in aversive shock stimulus (AS) presentation. Distance and attack speed were manipulated to be distant (easily escapable), imminent (with effort escapable at chance level), or inescapable, and all threat conditions (yellow pictures with shock hazard icon) were compared to an equivalent control condition (blue pictures with neutral icon) but without the threat of AS exposure. Note that this timing adjustment renders escape reaction time an uninformative behavioral measure, but it ensures that our measure of interest, acoustic startle reflex (ASR), is unaffected by the participant’s general ability in reaction speed. (B) During the anticipation phase, ASR was measured. See STAR Methods and Figure S1. (C) Estimated marginal means of the three-way—condition (threat and safe), distance (distant, imminent, and inescapable), group (BLA-damaged and healthy control)—interaction (Wald *χ*^*2*^ = 10.023; p = 0.040) of ASR magnitudes in the TET (BLA damage, n = 5; HC, n = 14). This interaction reveals reliable threat potentiation in imminent (Wald *χ*^*2*^ = 29.972; p < 0.001) and inescapable (Wald *χ*^*2*^ = 42.270; p < 0.001), but not in distant (Wald *χ*^*2*^ = 2.003; p = 0.157), conditions. Crucially, imminent threat potentiation was significantly stronger in BLA-damaged subjects (Wald *χ*^*2*^ = 6.191; p = 0.013) and, although HCs showed significantly lower threat potentiation in imminent compared to inescapable conditions (Wald *χ*^*2*^ = 4.670; p = 0.031), this was not the case in BLA-damaged subjects (Wald *χ*^*2*^ = 0.196; p = 0.658). ^∗^p < 0.05; ^∗∗^p < 0.01; ^∗∗∗^p < 0.001; see Table S2 for corresponding potentiation values and confidence intervals. Error bars represent standard error of the mean.

**Figure S1:**
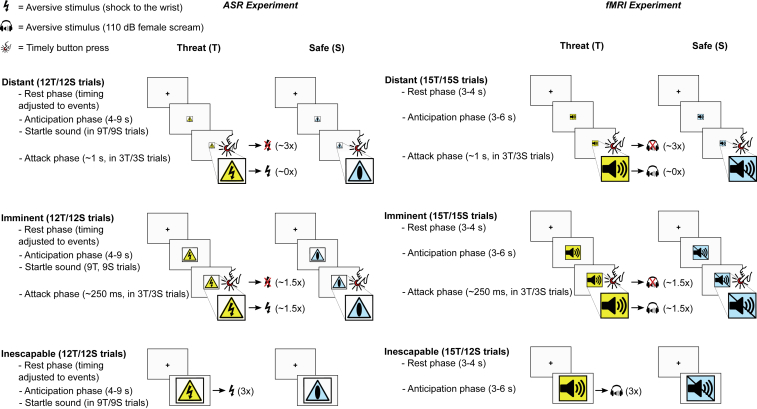
Outline of the TET for Both Human Experiments, Related to Figure 1 Participants are repeatedly attacked by rapidly approaching pictures, which they can escape from by pressing a button. When they fail to do so they will be presented with an aversive stimulus (AS). The speed of attack is manipulated to be distant-escapable, imminent (with effort escapable at chance-level) or inescapable, and all conditions are compared to an equivalent safe-context control condition involving the same procedure but without the threat of AS exposure.

We then examined whether this damage affected the interplay of active escape and passive defensive behavior. We compared UWD subjects with a healthy control (HC) group (matched for sex, age, IQ, and socioeconomic environment; Table S1) in an experimental environment wherein threat and escape possibilities dynamically changed. This “threat escape task” (TET) consisted of an aversive stimulus (an electric shock to the wrist) that could be avoided by pressing a button when a visual stimulus apparently approached (growing in size on screen; Figures 2A and S1). These “attacks” took place either under “distant” threat (small-sized visual stimulus = shock easily avoidable), “imminent” threat (medium size = shock with effort avoidable at 50% chance level due to online individually adjusted timing), or “inescapable” threat (full size = shock unavoidable). As readout of passive threat reactivity, we measured, during anticipation of these attacks, the acoustically triggered eye-blink startle reflex (ASR) (Figure 2B) using electromyography of the orbicularis eye muscle (Davis, 2006). ASR is typically potentiated in inescapable threat compared to safe conditions (Davis, 2006, Löw et al., 2015), and accordingly, we compared ASR between threat and safe trials (Figures 2A and S1). Threat and safe conditions were unambiguously communicated by means of a different visual stimulus and were, apart from the presence (threat) or absence (safe) of threat of shock, exactly similar.

In healthy controls, the increasing threat imminence (distant → imminent → inescapable) evoked, as expected, increasing potentiation of ASR compared to congruent safe conditions (Figure 2C; Table S2). A crucial difference appeared, however, between healthy controls and BLA-damaged subjects during exposure to the imminent threats, that is, during the condition where defensive freezing reactions have to be suppressed to allow successful escape (Löw et al., 2015, Moscarello and LeDoux, 2013). In BLA-damaged subjects, the ASR was potentiated by imminent threat to the same level as inescapable threat, whereas this potentiation by imminent threat was significantly lower in healthy controls (Figure 2C). This striking difference in potentiated startle between BLA-damaged human subjects and controls provides important first evidence that, while preparing for rapid escape from threat, a functional BLA inhibits reflexes associated with passive defensive behavior.

### BLA Regulates Pons-Driven Defensive Reflexes via the CeA

**Figure 3:**
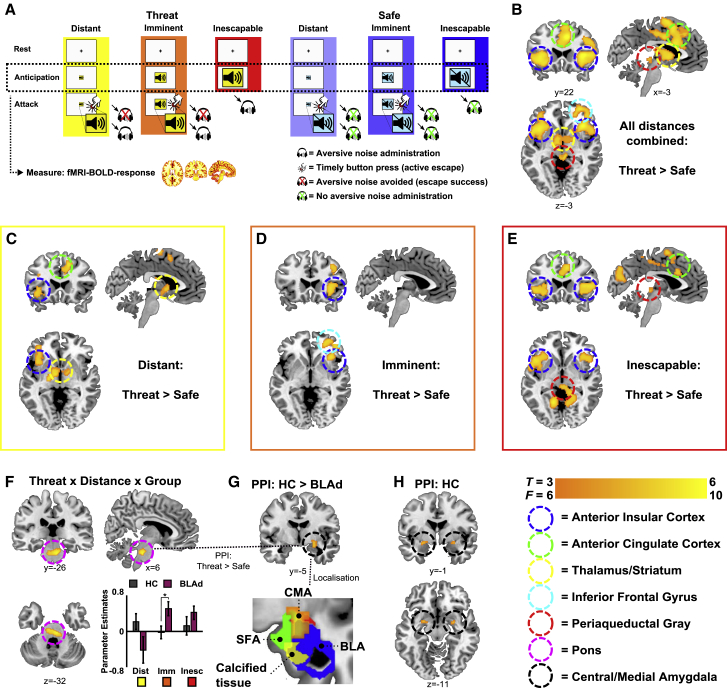
BLA Damage Leads to Increased Pons Reactivity to Imminent and Inescapable Threat Underpinned by Reduced Pons-CeA Coupling (A) Brief overview of the TET (see also Figure 2A). fMRI-blood oxygen level dependent (BOLD) is specifically modeled to measure brain activity during the anticipation phase. See STAR Methods and Figure S1. (B) General threat contrast across all participants (n = 20; t test: threat > safe). (C–E) Testing the threat distances separately revealed that the frontal areas of the salience network (anterior insula and anterior cingulate cortex) reliably increased activation at each distance (C), the right inferior frontal gyrus responded to imminent-escapable threat (D), and the PAG was only active in response to inescapable threat (E). See also Table S3. (F) Three-way interaction effect (*F*-test) of threat (threat and safe), distance (escapable, imminent, and inescapable), and group (BLA damaged [BLAd], n = 5; healthy controls [HCs], n = 15) defining a pontine cluster reactive to threat distance only in the BLAd group. Non-parametric tests revealed that activity (extracted parameters estimates; see bar graph) was significantly higher in the BLAd compared to HC group in the imminent threat condition (*Z* = 2.14; ^∗^p = 0.032). This comparison reached borderline significance (*Z* = 1.70; p = 0.089) in the inescapable threat and was non-significant in the distant threat condition (*Z* = −1.53; p = 0.127). Error bars represent standard error of the mean. (G) A 6-mm sphere around the peak voxel in this pontine cluster was used for a psycho-physiological interaction (PPI) analysis, identifying a cluster in the right CeA showing more threat-related connectivity with the pons in the HC compared to BLAd group. As shown, this cluster did not overlap with the location of BLA calcification. (H) PPI effect in the HC group, which illustrates that the group effect is due to a threat-specific increase in connectivity between the pons and CeA that was only observed in the HC group. All statistical parametric maps show significant clusters after family-wise error (FWE) correction (p < 0.05) except for (H), which was included for illustrative purposes (see Table S3 for FWE-corrected statistics). X, Y, and Z values indicate MNI coordinates, and non-thresholded statistical maps can be found at https://neurovault.org/collections/KEFBYYQG/.

We next set out to assess the pathway by which the BLA impacted the concomitant decrease in passive response to imminent escapable threat. Freezing and threat-potentiated startle responses are mediated through projections from the CeA to the brainstem, that is, to the periaqueductal gray (PAG) and the pons, respectively (Davis, 2006, Hermans et al., 2013). We first searched in the human subjects for activity changes in these areas by combining the TET with fMRI (Figure 3A).

In line with our previous work (Montoya et al., 2015) and instructed threat research in general (Mechias et al., 2010), the TET evoked threat reactivity across the brain’s salience network (anterior insula, anterior cingulate cortex, thalamus, and midbrain/PAG; Figures 3B–3E; Table S3). Crucially, we observed within the pons of the BLA-damaged subjects a cluster of voxels that was significantly more sensitive to threat distance than in healthy controls (Figure 3F; Table S3). This group difference was due to increased reactivity to imminent and inescapable threat, relative to distant conditions, and most pronounced in response to imminent threat (Figure 3F; Table S3). Thus, similar to the startle response, the pontine area of the brainstem showed a hyper-reaction to imminent threat conditions. Interestingly, in the HC subjects, this area of the pons showed threat-related functional connectivity with the CeA, which was significantly reduced in the BLA-damaged subjects (Figures 3G and 3H; Table S3). This finding suggests that hyper-reactivity observed in the pons of the BLA-damaged subjects is due to suboptimal regulation by the CeA. These results indicate that, particularly in situations of preparation for rapid escape, the BLA is critically involved in downregulation of pons-driven defensive reflexes through a mechanism that may involve an inhibitory network within the CeA.

### BLA Neuronal Silencing Induces Passive Defensive Reactions upon Imminent Threat

**Figure 4:**
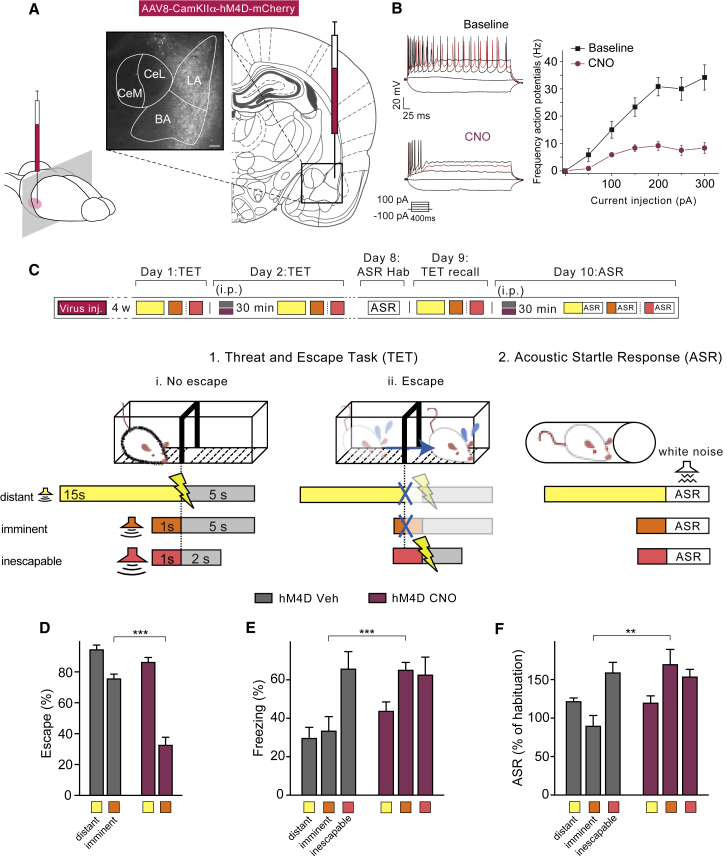
BLA Neuronal Downregulation Induces Passive Defensive Reactions upon Imminent Threat (A) Virus injection site and expression of hM4D revealed by mCherry immunohistochemistry. Scale bar, 200 μM. (B) (Left) Example traces of action potentials fired during 400-ms incremental current injections (from −100 pA to 300 pA; red trace representing 200 pA; see inset) before (top) and during CNO treatment (bottom). (Right) Mean action potential frequency as function of current injected before CNO (black) and during CNO (purple) is shown. (C) Experimental design for the TET and ASR assessment: day 1, TET conditioning; day 2, TET testing with vehicle (n = 6, gray) or CNO (n = 7, purple) intraperitoneal (i.p.) injected in the BLA 30 min prior to (1) exposure in threat and escape task (TET) to distant (4 kHz, yellow), imminent (12 kHz, orange), or inescapable (12 kHz, red; in separate experiment) threat. (i) “No escape” illustrates shuttling only upon foot shock exposure; (ii) “escape” illustrates avoidance of foot shock by shuttling before end of tone. (2) Acoustic startle response measured after exposure to 4- or 12-kHz tones followed by white noise burst on day 8 (ASR habituation), followed on day 9 by TET recall, and on day 10 by ASR 30 min after vehicle (gray) or CNO (purple) i.p. injection. (D and E) CNO (purple) as compared to vehicle (gray) i.p. injections in hM4D-infected rats that were exposed to imminent threat (D) reduced escape responses (two-way ANOVA: treatment × threat imminence; F_(1, 22) =_ 19.48; p < 0.001) and (E) increased freezing levels (two-way ANOVA: treatment effect; *F*_(1, 22) =_ 16.69; p < 0.001). Imminent threat induces significantly more freezing compared to distant threat in rats injected with CNO compared to Veh (imminence effect *F*_(1, 22)_ = 5.06; p < 0.05). CNO has no effects on freezing after inescapable threats. (F) CNO enhanced the potentiation of the startle reflex (ASR; two-way ANOVA: treatment × threat imminence; *F*_(1, 20)_ = 10.21; p < 0.01; n = 6 each group) that occurs upon exposure to imminent, but not to distant or inescapable, threats. Data from TET test conducted under naive conditions and vehicle treatments were pooled and converted to percent escape behavior and were normally distributed (D’Agostino and Pearson normality test; for distant threat, K_2_ = 5.40, p = 0.06; for imminent threat, K_2_ = 1.11, p = 0.57). ^∗∗^p < 0.01; ^∗∗∗^p < 0.001. Error bars represent standard error of the mean. See also Figures S2 and S3.

**Figure S2:**
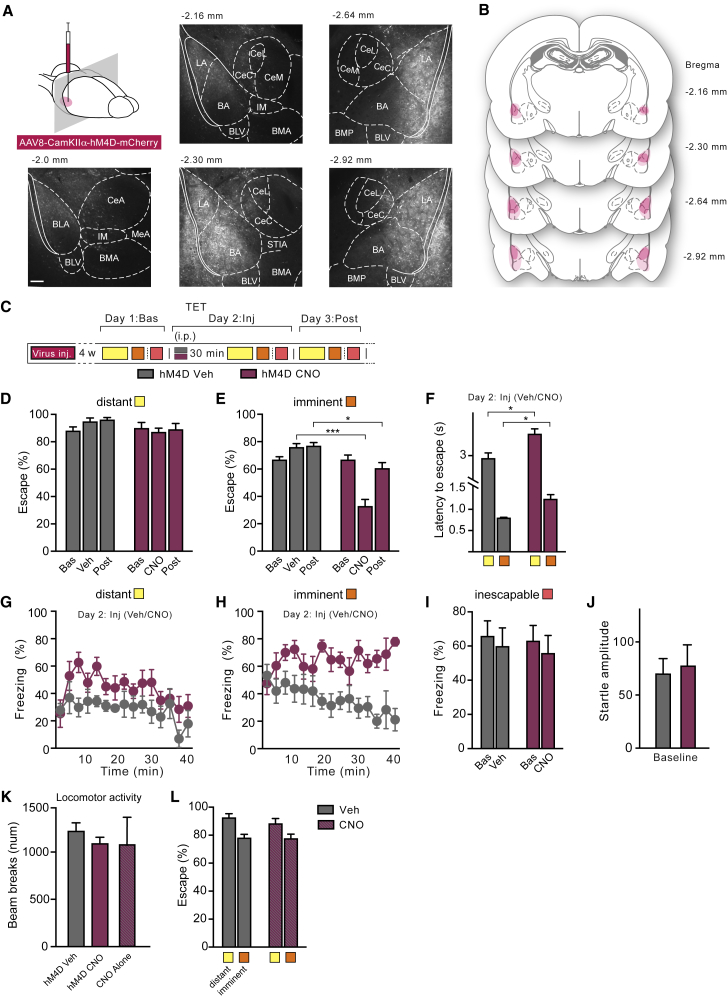
Combining TET with Chemogenetic Inhibition of BLA, Related to Figure 4 (A) Representative examples of viral expression in the BLA (in rostro-caudal order) after injection with AAV-CaMKIIα-hM4D-mCherry. Viral expression frequently extended somewhat beyond the LA laterally and dorsally and included parts of the dorsal endopiriform nucleus. (B) Pink areas represent the minimum (darker color) and the maximum (lighter color) expression of AAV-CaMKIIα-hM4D-mCherry (C) Four weeks after virus injection rats were conditioned on day 1 in the TET to establish baseline value (“Bas”) and then tested on day 2 (“Inj”) 30 min after Vehicle (“hM4D Veh,” gray) or CNO injection (“hM4D CNO,” orange). (D) We found no differences between groups in baseline escape to distant threats. downregulation of the BLA did not affect escape responses to distant threat. (E) Decrease of escape response to imminent threat after BLA inactivation (two-way RM ANOVA: treatment x time effect, *F*_(2, 22)_ = 24.29, p < 0.001). After 24 hours from injection (post), this effect was still present to a lesser extent. No baseline differences between groups in escape responses to imminent threat. (F) CNO injection increased latency to escape both to distant (unpaired t test, t = 0.01 df = 11, p = 3.01) and imminent (unpaired t test, t = 0.006 df = 11, p = 3.31) threats (G) Time course showing that over several trials BLA inactivation did not affect freezing reaction to distant threat. (H) Time course overall several trials showing that downregulation of the BLA increased freezing levels to imminent threat (two-way RM ANOVA: treatment x time effect, *F*_(14, 154)_ = 2.88, p < 0.01). (I) CNO did not induce any change of freezing behavior after the presentation of inescapable threats *(F*_(1, 10)_ = 0.01, p = 0.90). (J) Baseline amplitude of startle reflex following Veh or CNO injection (unpaired t test, t = 0.29 df = 10, p = 0.77). ^∗^p < 0.05, ^∗∗^p < 0.01, ^∗∗∗^p < 0.001 (K) CNO did not induce any gross locomotor deficit in hM4D-injected and naive rats, compared to Veh rats (*F*_(3, 22)_ = 0.826, p = 0.85). (L) CNO treatment in naive rats did not induce any impairment in escape behavior to distant and imminent threats compared to veh-treated rats (two-way ANOVA: treatment, *F*_(1, 18)_ = 0.49, p = 0.48).

To assess directly whether the BLA has a functional role in controlling responses to imminent threat, we translated these human experiments into a rodent model. We therefore chemogenetically targeted glutamatergic neurons in the rat BLA with a virus carrying the inhibitory designer receptors exclusively activated by designer drugs (DREADD) receptor hM4Di (Figures 4A, S2A, and S2B), an engineered inhibitory G-protein-coupled receptor that can decrease neuronal activity. Notably, activation of hM4Di by clozapine-N-oxide (CNO), the agonist of hM4Di (Alexander et al., 2009), reversibly induced membrane hyperpolarization and decreased neuronal firing (Figure 4B), which indicates that activity of BLA neurons was temporarily attenuated.

**Figure S3:**
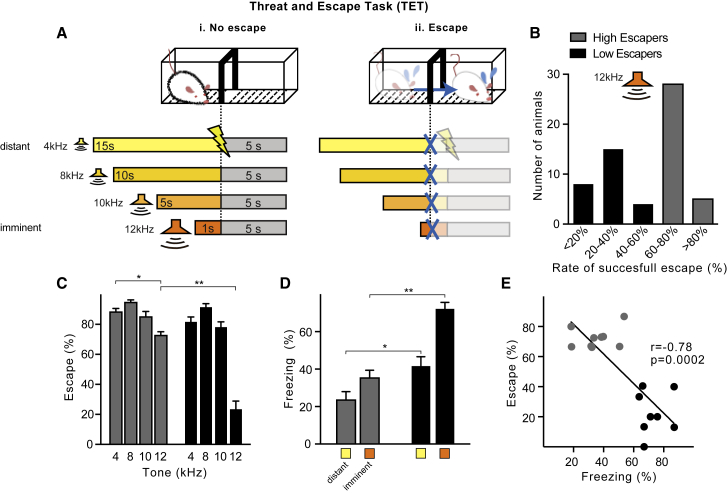
Trait Variability in Rats in the TET, Related to Figure 4 (A) Threat and Escape Task (TET) protocol: in a two-way shuttle box, rats were conditioned to tones announcing an electric shock that could be avoided by moving to the other compartment before the end of the tone. Animals were trained over 5 days to different tones of increasing frequency/decreasing duration (4 kHz/15 s, 8 kHz/10 s, 10 kHz/5 s and 12 kHz/1 s). Thus, higher frequencies were associated to higher threat imminence that required more rapid escape responses. 1. In case of ‘No escape’ rats received an electric shock, that could be turned off by shuttling to the other compartment, while after an 2. ‘Escape’ response the tone was turned off until the next trial. See STAR Methods for more details. (B) Population of rats tested in this study. Rats diverged in their coping strategies in response to imminent threats. About half adopted an active coping by escaping more than 60% of the trials (“high escapers” or HE), while the rest adopted a passive coping strategy (“low escapers” or LE). (C) Naive rats showed decreased escape efficiency to imminent (12 kHz/1 s) compared to distant threat (4 kHz/15 s; at least p < 0.05), however, low escapers performed significantly poorer compared to high escapers (two-way ANOVA: imminence x group effect, *F*_(3, 60)_ = 23,01, p < 0.001; n = 8-9 each group). (D) Low escapers showed naturally higher freezing reactions upon presentation of the distant (4kHz) and imminent (12kHz) conditioned tones (two-way ANOVA: imminence x group effect, *F*_(1, 30)_ = 4,61, p < 0.05). (E) Within-group correlations between escape and freezing behaviors upon exposure to imminent threat (12 kHz/1 s). An inverse correlation (*r* = −0.78, p = 0.0002) emerged suggesting that expression of these behaviors was co-regulated.

We then developed a TET equivalent for rats (Figures 4C, S2, and S3), expanding upon the traditionally used single-compartment, inescapable-threat task, by adding an adjacent compartment for escape. In this two-compartment shuttle box, we conditioned rats to tones announcing an electric shock that could be avoided by shuttling to the other compartment before the end of the tone. Successive exposure to tones of decreasing duration but increasing frequency (ranging from 15 s/4 kHz to 1 s/12 kHz) allowed the rats to associate higher tones with higher threat imminence that required more rapid escape.

After bilateral injection of AAV-CamKIIα-hM4D-mCherry in the BLA, we trained rats in the TET (Figures S2 and S3) and assessed their baseline level of escape to distant versus imminent threats. We identified two sub-populations of rats, hereafter called high escapers, or “HE,” and low escapers, or “LE,” based on their different behaviors when presented with imminent threats (Figure S3). In particular, HE rats showed significantly higher escape successes and less freezing reaction to imminent threats compared to LE rats (Figure S3). We then selected HE rats and treated them with vehicle or CNO for BLA neuronal silencing. Whereas CNO-induced downregulation of the BLA did not affect responses to distant (4 kHz) threats, it significantly decreased escape performance to imminent (12 kHz) threats, as compared to vehicle treatment (Figure 4D). Moreover, this effect was temporary and reversible as, 24 hr after CNO injection, rats recovered baseline levels of escape behavior (Figure S2E). Escape behavior and locomotor activity were not affected by CNO treatment in virus-uninfected rats (Figure S2). Upon presentation of the imminent threat to rats with chemogenetical downregulation of the BLA as compared to vehicle-treated rats, we also observed increased freezing and, similar to our findings in BLA-calcified humans, enhanced potentiation of the startle response. Upon presentation of distant or inescapable threats, CNO-treated rats showed similar freezing levels and potentiation of the startle response as compared to vehicle-treated rats (Figures 4E and 4F). Taken together, these results show that, when a threat becomes imminent in rodents, the BLA is needed to decrease freezing and startle and direct behavior toward rapid escape.

### Successful Conditioning to Escape from Imminent Threat Potentiates BLA Projections to a Subset of CeL Neurons

We then set out to uncover the mechanism through which the BLA induced a switch in behavior between passive and active responses to imminent yet escapable threat. In mice, stimulation of glutamatergic terminals that originate from the BLA has recently been shown to activate neurons in the CeL that can inhibit CeM neurons and reduce freezing responses (Tye et al., 2011). In the rat CeL, we have previously identified such a group of neurons: their direct activation by oxytocin inhibits freezing through GABAergic projections onto CeM neurons that further project to the ventrolateral PAG (Huber et al., 2005, Tovote et al., 2016, Viviani et al., 2011). We therefore focused our further experiments in rats on oxytocin-sensitive neurons as candidate targets of the BLA.

**Figure 5:**
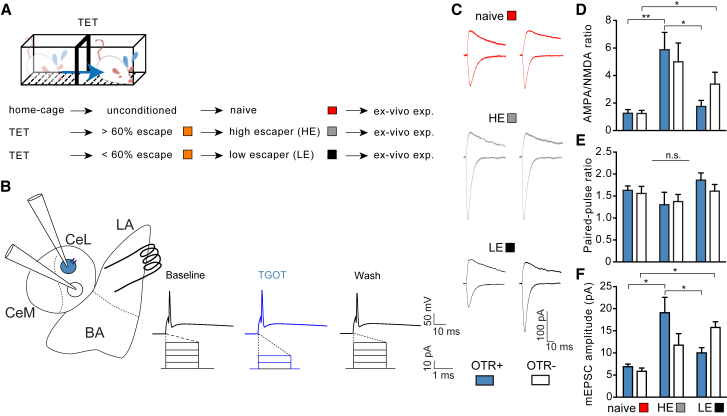
Potentiation of BLA Projections onto OT-Sensitive CeL Neurons after TET (A) We used rats tested in TET and divided into high (HEs) (gray) and low escapers (LEs) (black) by, respectively, their escaping more or less than 60% of the imminent trials (orange; see Figure S3 for details). Non-TET-trained rats (“naïve,” red) served as additional controls. (B) Schematic coronal rat amygdala slice with stimulation and recording electrodes placed on neurons sensitive (blue) or not (white) to TGOT as revealed by TGOT-reducing level of current injection needed to evoke action potential. (C) Representative evoked EPSC traces recorded from OTR+ and OTR− cells in slices of the CeL of rats as coded in (A). (D) High-escaper rats after TET conditioning showed higher AMPA/NMDA ratio in OTR+ cells in the CeL compared to low escapers (n = 49, 10 rats; two-way ANOVA: group effect; *F*_(2, 43)_ = 13.17; p < 0.0001). ^∗^p < 0.05; ^∗∗^p < 0.001. (E) No effect of TET conditioning on paired-pulse ratio (n = 43, 10 rats; *F*_(2, 35)_ = 0.38; p = 0.68). (F) mEPSCs obtained in the CeL in presence of tetrodotoxin showed higher amplitude in OTR+ cells in HE compared to naive and LE rats (n = 48, 10 rats; two-way ANOVA: group × treatment effect; *F*_(2, 42)_ = 4.30; p < 0.01). ^∗^p < 0.05. Error bars represent standard error of the mean. See also Figure S4.

**Figure S4:**
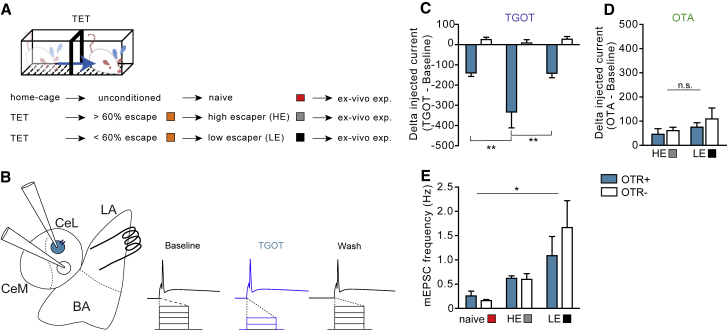
CeA Cells Sensitivity to OT Agonist, Related to Figure 5 (A) We conditioned naive rats in the TET which, depending on the coping strategies in response to imminent threats, were classified in two groups: high-escapers (adopted an active coping by escaping more than 60% of the imminent trials) and low-escapers (escaped less than 60% and displayed significantly higher freezing behavior, see Figure S3 for details). Additionally, for electrophysiological experiments we included a group of naive, unconditioned rats. Brains were collected about 30 min after the end of the session for the preparation of acute coronal slices containing the LA and CeL. (B) We identified OTR-positive (in light blue) and -negative cells (in white) by application of selective agonist TGOT. In current clamp, a protocol of 20 steps of current (10pA) was employed, to observe the current threshold to elicit an action potential in the patched cells. For each cell, the protocol was run in basal conditions (ACSF), during TGOT (1μM) infusion, and wash 20 min. after. If the cell experienced a reversible decrease of the current required for firing, it was considered as OTR+. Square pulses illustrate increasing current injection. (C) We found a higher sensitivity of OTR+ cell to TGOT in HE rats compared to naive unconditioned and LE rats (n = 101, 18 rats; one-way ANOVA: *F*_(2, 95)_ = 4.03, p < 0.05). (D) No differences in sensitivity to OTA between HE and LE rats (two-way ANOVA: *F*_(1, 20)_ = 0.11, p = 0.73). (E) Increase frequency of mEPSCs in LE rats as compared to naive rats (n = 10 rats; two-way ANOVA: group effect, *F*_(2, 42)_ = 6.62, p < 0.01). ^∗^p < 0.01

Our next step was to identify projections from the BLA to the CeL that could play a role in behavioral responses to imminent escapable threat. For this purpose, we measured changes in BLA-induced excitatory synaptic transmission *in vitro* onto different types of CeL neurons after escape conditioning (Figure 5A). We first trained a new cohort of naive rats on the TET and prepared *in vitro* brain slices from these animals. In the CeL, we then used whole-cell patch-clamp recordings to measure excitatory postsynaptic currents (EPSCs), evoked by a stimulating electrode placed in the lateral part of the BLA (Figure 5B). Previous findings in these synapses had shown, after classical threat conditioning, an increase in *α*-amino-3-hydroxy-5-methyl-4-isoxazolepropionic acid (AMPA)/N-methyl-D-aspartate (NMDA) ratio, an index of glutamatergic synaptic strength, as a result of synaptic potentiation of BLA-CeL connections onto SOM+ neurons (Li et al., 2013). Thus, we assessed potentiation of inputs by measuring responses mediated by both AMPA and NMDA receptors in individual cells. We categorized different neurons in acute rat coronal slices of the CeL by their sensitivity to the specific, bath-applied, oxytocin receptor agonist (Thr^4^,Gly^7^)-oxytocin (TGOT) (Figure 5B). To identify changes in transmission underlying responses to imminent escapable threat, we compared changes in BLA-CeL transmission between HE and LE rats, which differ in their development of successful escape responses specifically to imminent escapable threat (Figures 5C and S3C). As can be seen in Figure 5D, in slices from HE rats, AMPA/NMDA ratios had significantly increased in TGOT-responsive neurons (OTR+) compared to the AMPA/NMDA ratios in TGOT-responsive neurons from LE or naive, unconditioned rats. We also observed a potentiation of the TGOT-unresponsive neurons (OTR−), but this potentiation did not differ between HE and LE rats. These findings thus pointed to the potentiation of projections onto TGOT-sensitive neurons as a hallmark that distinguished the efficient learning of escape from imminent threat. We observed no changes in paired-pulse ratios between any of the groups (Figure 5E) but significant changes in amplitudes of miniature EPSCs (mEPSC) that matched the changes found in the AMPA/NMDA ratio (Figures 5E and S4E), indicating that the observed changes in synaptic strength were the result of a postsynaptic mechanism.

### OTR+ Neurons Modulate the Switch between Active and Passive Responses to Imminent Threat

**Figure 6:**
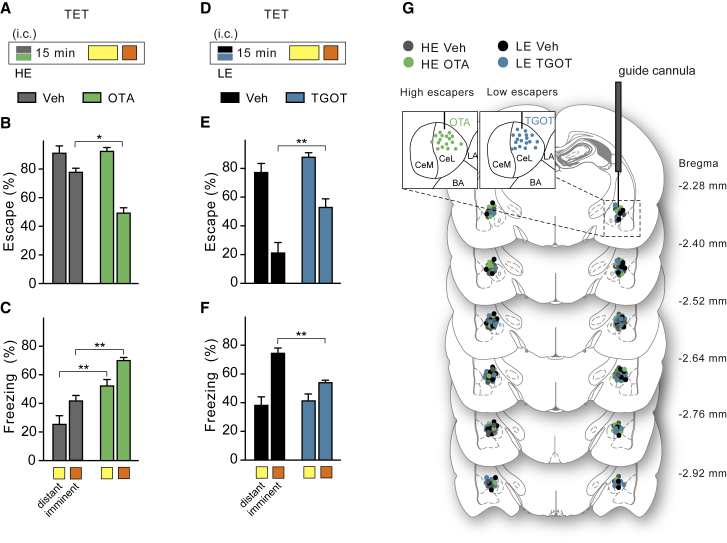
Oxytocin-Sensitive Neurons Modulate the Switch between Active and Passive Responses to Imminent Threat (A) HE rats received injections with vehicle (gray) or OTA (green) that were targeted through cannulae intracerebrally (i.c.) into the CeL. 15 min later, both groups were tested for responses to distant threats (4 kHz, yellow) and imminent threats (12 kHz, orange) in the TET. (B) OTA in HE rats reduced escape to imminent, but not distant, threat (two-way ANOVA: treatment × imminence *F*_(1, 22)_ = 16.57; p < 0.001). ^∗^p < 0.05. (C) OTA in HE rats increased freezing both to distant and imminent threat (two-way ANOVA: imminence effect *F*_(1, 18)_ = 13.73, p < 0.01; treatment effect *F*_(1, 18) =_ 36.30, p < 0.001; n = 6–7 each group). ^∗∗^p < 0.01. (D) LE rats received injections with vehicle (black) or TGOT (blue) that were targeted through cannulae intracerebrally (i.c.) into the CeL. 15 min later, both groups were tested for responses to distant threats (4kHz, yellow) and imminent threats (12 kHz, orange) in the TET. (E) TGOT increased escape to imminent, but not distant, threat (two-way ANOVA: imminence effect *F*_(1, 22)_ = 55.36, p < 0.001; treatment effect *F*_(1, 22)_ = 2.95, p < 0.01). ^∗∗^p < 0.01. (F) TGOT decreased freezing to imminent, but not distant, threat (two-way ANOVA: imminence × treatment effect *F*_(1, 20)_ = 7.27, p < 0.05; n = 6–7 each group). ^∗∗^p < 0.01. (G) Localization of microinjector tips for each animal according to brain atlas of Paxinos and Watson (1997). Error bars represent standard error of the mean. See also Figure S5.

**Figure S5:**
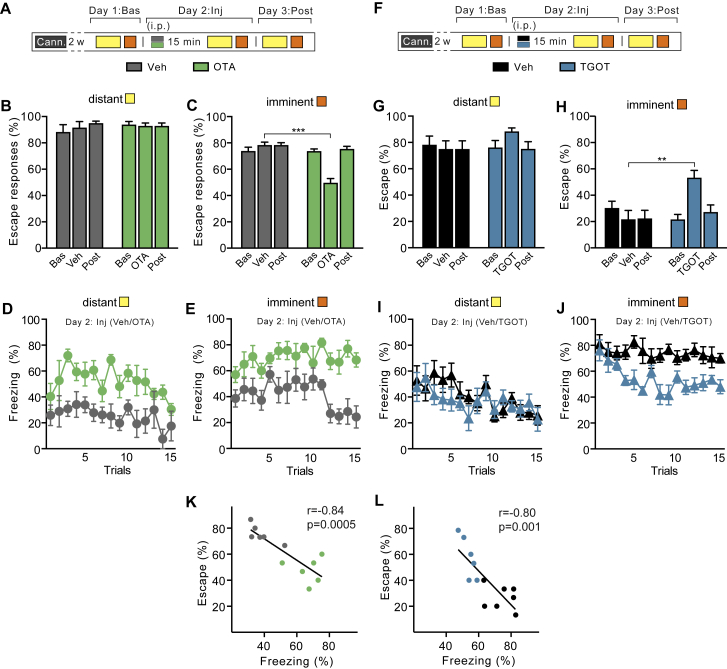
OTR+ Neurons Modulate the Switch between Active and Passive Responses to Imminent Threat, Related to Figure 6 (A) Behavioral procedure - TET protocol. Rats were implanted bilaterally with guide cannulae above the CeL. After 2 weeks of recovery, rats were conditioned in the TET to determine baseline escape performance (“Day 1: Bas”). The following “Day 2: Inj” rats were tested 15 min after Veh or OTA (i.c.) infusion in the CeL. After 24 hours rats were re-tested without any treatment (“Day 3: Post”). (B) No differences in escape responses to distant threat. (C) Bilateral OT receptors blockade (OTA) in the CeL reduced escape responses to imminent threat compared to Veh animals (two-way RM ANOVA: treatment x time, *F*_(2, 22)_ = 18.70, p < 0.001). Rats did not differ in baseline escape behavior nor after 24 hours washout. (D) Time course of freezing reaction to distant threat, following Veh or OTA infusion (two-way RM ANOVA: treatment x time, *F*_(1, 9)_ = 11.51, p < 0.05). (E) Time course of increased freezing responses to imminent threat following OTA infusion compared to Veh (two-way RM ANOVA: treatment, *F*_(1, 9)_ = 37.62, p < 0.001). (F) Behavioral procedure - in a subgroup of rats that showed natural low escape performance and high levels of freezing (“low escapers” or LE) we tested OT receptor agonist in the CeL. (G) We found no differences in escape responses to distant threat. (H) activation of OT receptors by TGOT in the CeL in LE rats significantly increased escape responses to imminent threat (two-way RM ANOVA: treatment, *F*_(2, 22)_ = 20.40, p < 0.001). (I) Time course of freezing levels upon exposure to distant threat, following Veh or TGOT infusion. (J) Time course of freezing levels showing when OT receptor activation in the CeL significantly reduced freezing responses to imminent threat in LE rats (two-way RM ANOVA: treatment, *F*_(1, 11)_ = 28.48, p < 0.001). (K and L) Escape and freezing behaviors to imminent threat are negatively correlated in HE rats after Veh or OTA (*r* = −0.84, p = 0.0005; n = 6 each group) and in LE rats after Veh or TGOT infusion in the CeL (*r* = −0.80, p = 0.0001; n = 6-7 each group). ^∗∗^p < 0.01, ^∗∗∗^p < 0.001

To further test a possible role of these oxytocin-sensitive neurons in responses to imminent threats, we pharmacologically manipulated their activity through cannulae bilaterally targeting the CeL in both HE and LE rats. In line with established inhibition of freezing by endogenous oxytocin (Knobloch et al., 2012), blocking their receptor with the specific oxytocin receptor antagonist [1-D(CH_2_)5,Tyr(ME)2,Thr^4^,Tyr-NH_2_(9)]ornithine vasotocin in the HE rats (OTA) (Figures 6A, 6G, and S5) effectively decreased escape performance to imminent threat (Figure 6B) and increased freezing both to distant and imminent threat (Figures 6C and S5). To test whether, conversely, direct activation of these neurons increased escape performance and decreased freezing, we tested LE rats which, at baseline, exhibited low escape performance and high freezing responses to imminent threat (Figure S3). Injection of the specific oxytocin receptor agonist TGOT in this group (Figures 6D and 6G) effectively increased escape performance and decreased freezing to imminent, but not distant, threat (Figures 6E and 6F). *Ex vivo* electrophysiological recordings after training revealed a higher sensitivity to TGOT by CeL neurons of HE compared to LE and naive rats (Figures S4C and S4D). Taken together, these findings further confirm a central role of OT-sensitive CeL neurons in promoting active responses to imminent threat.

### Activation of OTR+ Neurons during Downregulation of the BLA Can Rescue the Lost Escape Responsiveness to Imminent Threat

**Figure 7:**
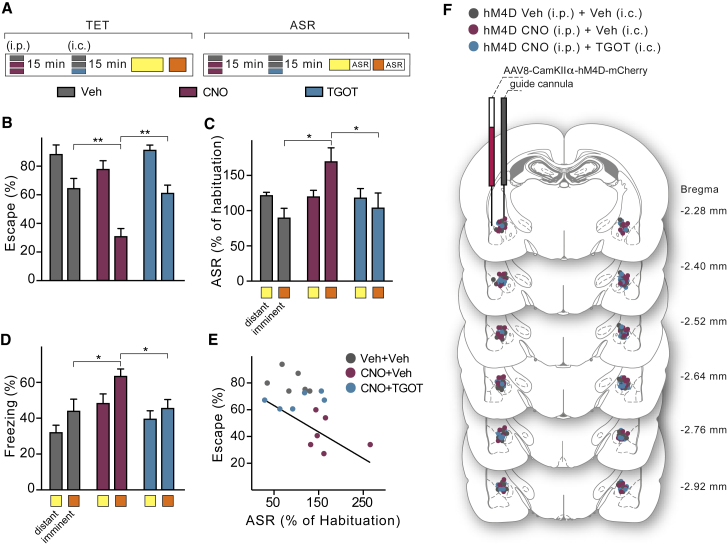
Activation of Oxytocin-Sensitive Neurons during Downregulation of the BLA Rescues the Switch between Active and Passive Responses to Imminent Threat (A) Rats treated with i.p. injection of vehicle (gray) or CNO to inhibit the BLA (purple) received 15 min later either vehicle (gray) or TGOT (blue) by i.c. injection into the CeL 15 min before tested in the TET. Vehicle and vehicle, n = 6; CNO and vehicle, n = 8; CNO and TGOT, n = 8. (B) TGOT rescued the escape deficit to imminent threat induced by CNO-mediated inactivation of the BLA (two-way ANOVA: imminence effect *F*_(1, 36)_ = 48.91, p < 0.001; treatment effect *F*_(2, 36)_ = 9.44, p < 0.001). Escape to distant threat was unaffected by CNO or TGOT. ^∗∗^p < 0.01. (C) TGOT abolished the potentiation of acoustic startle to imminent threat obtained by CNO-induced inactivation of the BLA (two-way ANOVA: imminence × treatment effect *F*_(2, 30)_ = 4.21; p < 0.05). Neither CNO nor TGOT affected startle potentiation by distant threat. ^∗^p < 0.05. (D) CNO-increased freezing to imminent threat reverted back to vehicle levels after TGOT (two-way ANOVA: imminence effect *F*_(1, 32)_ = 7.02, p < 0.05; treatment effect *F*_(2, 32)_ = 6.89, p < 0.01). Neither CNO nor TGOT affected freezing to distant threat. ^∗^p < 0.05. (E) Potentiation of the acoustic startle to imminent threats was inversely correlated to escape proficiency (*r* = −0.58; p = 0.01; n = 18). (F) Localization of microinjectors tips for each animal according to brain atlas of Paxinos and Watson (1997). Error bars represent standard error of the mean. See also Figure S6.

**Figure S6:**
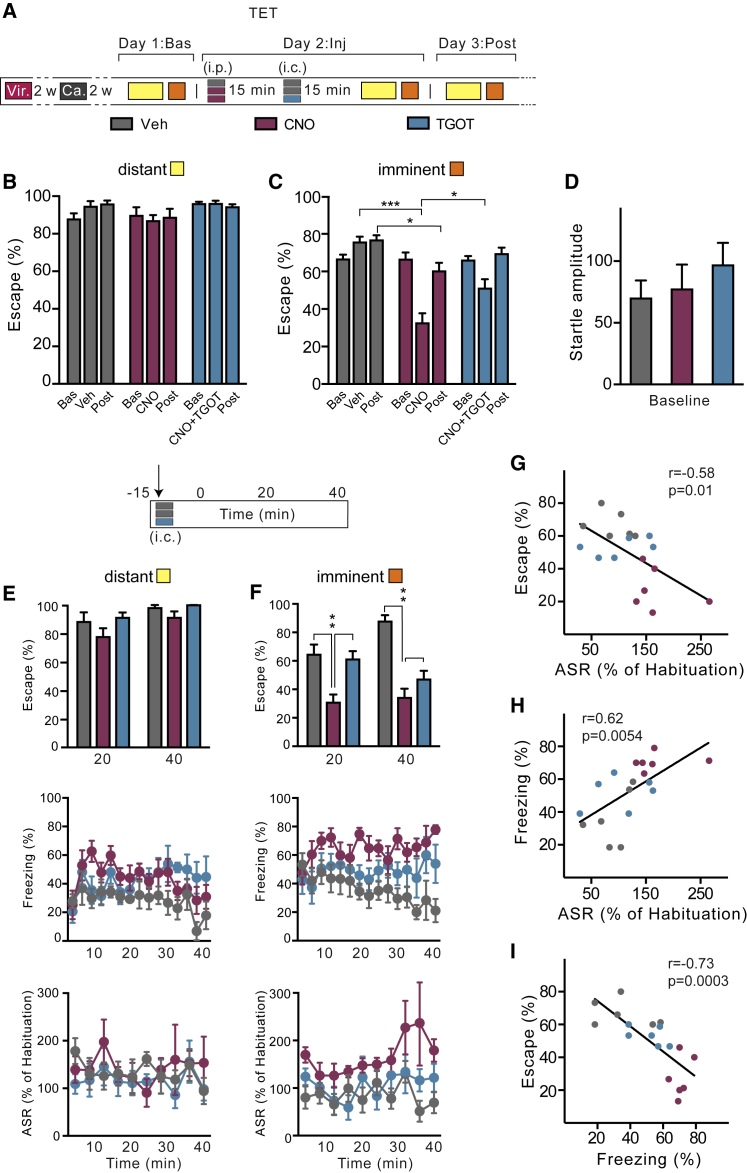
Activation of OTR+ Neurons during Downregulation of the BLA Can Modulate the Switch between Active and Passive Responses to Imminent Threat, Related to Figure 7 (A) Behavioral procedure – TET protocol. Two weeks after virus injection (AVV_8_-CamKIIα-hM4D-mCherry) rats were implanted bilaterally with guide cannulae above the CeL. After 2 weeks of recovery, rats were conditioned in the TET to establish baseline values (Day 1: Bas). The following Day 2: Inj. three groups of animals were tested (n = 6-8 rats per group): “hM4D Veh (i.p) + Veh (i.p.)” (gray) received vehicle injections only; “hM4D CNO (i.p.) + Veh (i.c.)” (orange) received CNO i.p. and after 15 min vehicle i.c.; “hM4D CNO (i.p.) + TGOT (i.c.)” (blue) received CNO injection i.p. and after 15 min. TGOT i.c. microinjection in the CeL. Subsequently they were tested in the TET. After 24 hours rats we re-tested without any treatment (“Day 3: Post”). (B) No differences in escape responses to distant threats. (C) Following BLA inactivation, bilateral TGOT infusion in the CeL increased escape responses compared to Veh animals (two-way RM ANOVA: treatment x time, *F*_(4, 36)_ = 10.12, p < 0.001). After washout (Post), escape behavior was still reduced in rats with inactivated BLA by CNO injection, but not in animals previously treated with TGOT. We observed no differences in baseline escape behavior to imminent threats. (D) Baseline startle amplitude as measured during habituation showed no differences between the three groups. (E) Because previous studies showed that TGOT has a temporally-limited effect, we grouped escape responses in blocks of 20 minutes (*top)*, and we show the time course over 40 minutes of freezing (*middle*) and ASR (*bottom*). We found no significant differences in escape responses, freezing and ASR to distant threat. (F) *Top*, rescue of escape behavior to imminent threat by TGOT infusion in the CeL following BLA inactivation, 20 min after infusion (two-way RM ANOVA: treatment x time *F*_(2, 18)_ = 3.79, p < 0.05). After 40 min TGOT had no effect on escape during concomitant BLA inactivation. BLA downregulation (hM4D CNO) reduced escape behavior during the entire session. *Middle*, time course over 40 minutes of freezing levels. CNO-mediated BLA inactivation increased freezing response (two-way RM ANOVA: treatment x time *F*_(28, 224)_ = 1.57, p < 0.05). *Bottom*, time course over 40 minutes of ASR. CNO-induced BLA inactivation increase ASR to imminent threats (two-way RM ANOVA: treatment effect *F*_(2, 15)_ = 5.97, p < 0.05). (G) Inverse correlation between escape responses and ASR to imminent threat in rats after Veh+Veh, CNO+Veh or CNO+TGOT injections (*r* = −0.58, p = 0.01; n = 6 each group). (H) Significant correlation between freezing and ASR (*r* = 0.62, p = 0.0054; n = 6 each group). (I) Significant correlation between escape responses and freezing (*r* = −0.73, p = 0.0003; n = 6 each group). ^∗^p < 0.05, ^∗∗^p < 0.01, ^∗∗∗^p < 0.001

Building on these findings, we tested whether activation of oxytocin-sensitive neurons in the CeL also rescued the changes induced by chemogenetic downregulation of the BLA (Figures 4D–4F). We therefore infused TGOT into the CeL of rats that, as a result of CNO downregulation of the BLA, had decreased escape performance and increased passive defensive responses (Figures 7A and 7F). TGOT in these rats increased escape to imminent threat to the same level as that in animals that had received vehicle instead of CNO and TGOT (Figures 7B and S6). Moreover, TGOT protected startle potentiation and freezing against the effects of CNO-induced BLA downregulation (Figures 7C, 7D, and S6). Throughout these experiments, escape performance exhibited an inverse correlation with ASR and freezing for all animals (Figures 7E and S6), suggesting a mechanism through which these two types of behaviors are oppositely regulated. These results suggest that, under conditions of reduced functionality of the BLA, activation of TGOT-responsive neurons in the CeL can restore correctly timed behavioral responses to the imminent threat stimulus.

## Discussion

We studied the function of the BLA in passive and active defensive behavior using a translational multi-method approach, in which we tested humans with bilateral BLA damage and rodents with a chemo-genetically silenced BLA. Our cross-species behavioral research reveals that, when rodents and humans are under imminent escapable threat, the BLA is essential for the selection and execution of rapid escape behavior. Through neuroimaging and neurobiological experiments, we next uncovered the mechanism by which the BLA implements rapid escape under imminent threat, namely by activation of a specific group of CeA neurons.

Our behavioral findings, showing increased startle potentiation under imminent escapable threat in both humans and rodents, laid the foundation for subsequent components of the study. Startle is a defensive reflex that is typically potentiated during inescapable threat but that is reduced when preparing for rapid active escape behavior (Löw et al., 2015). The main finding of our study is that, after BLA damage or silencing, threat potentiation of startle remains fully intact, but rapid-escape-related startle reduction fails. Furthermore, together with a lack of startle reduction during imminent escapable threat, BLA-silenced rats showed maladaptive freezing as well as reduced escape performance, indicating that the BLA is necessary for a switch from passive defensive to active escape behavior. Correspondingly, our neuroimaging data showed that, under imminent threat, BLA-damaged humans have abnormally high activity in the pontine brainstem, a region that plays a key role in initiating the startle reflex (Davis et al., 1982). Exploratory functional connectivity analyses on these human neuroimaging data furthermore suggested that the BLA acts via the CeA. *Ex vivo* electrophysiological and *in vivo* pharmacological measurements in rats indeed found that projections from the BLA onto oxytocin-sensitive neurons in the CeL are potentiated after successful conditioning to imminent yet escapable threat and that these neurons play a central role in downregulating freezing and fostering rapid escape. These findings not only show that the BLA, in concert with the CeL, is essential for rapid escape behavior but also indicate that this function of the BLA is conserved across rodents and humans.

The adaptive switch from passive to active defensive responses to imminent threat is compatible with the known anatomy of BLA projections that target different cell types in the CeL. Previous work in mice has shown a group of neurons in the CeL that, upon activation by the BLA, decreases neuronal activity in the CeM and concomitant anxiety-like behaviors (Tye et al., 2011). Our previous *in vitro* and *in vivo* work in the CeA has also identified a group of neurons in the CeL with inhibitory projections to the CeM and where activation by OT rapidly and reversibly reduces conditioned freezing behavior (Huber et al., 2005, Viviani et al., 2011). Our findings are thus consistent with the hypothesis that potentiation of BLA projections onto OTR+ neurons in the CeL, by suppression of freezing, allows escape from imminent threat.

In addition to OTR+ neurons, two other functional groups of neurons have been described in the mouse CeL. Fadok et al. (2017) identified a group of corticotropin-releasing factor (CRF)-expressing neurons (CRF+) where optogenetic activation was able to trigger non-oriented escape responses. Ciocchi et al. (2010) and Haubensak et al. (2010) have described somatostatin-expressing neurons (SOM+), where optogenetic activation instead disinhibited neurons in the CeM and increased freezing through activation of projections to the ventrolateral PAG (Fadok et al., 2017, Huber et al., 2005, Tovote et al., 2016, Viviani et al., 2011). Li et al. (2013) showed an increase of synaptic strength from BLA inputs onto these SOM+ neurons, concomitant with decreased synaptic input onto SOM− neurons after passive threat conditioning, i.e., following a conditioning protocol that did not allow for escape from the threat. It is therefore possible that the potentiation that occurs after escape from imminent threat training on the insensitive OT (OTR−) neurons, in both HE and LE rats, involves the SOM+ and CRF+ neurons. These might underlie the observed freezing responses before and after escape behavior, respectively, the learning of escape during distant or inescapable threat. Indeed, avoidance behavior is considered a sequential process in which the subject first rapidly learns an association between a conditioned stimulus and an aversive unconditioned stimulus through Pavlovian threat conditioning, which importantly depends on the BLA in both rodents and humans (Klumpers et al., 2015, LeDoux et al., 2017). This first Pavlovian stage is followed by a negative reinforcement process that guides the instrumental acquisition of the avoidance response (LeDoux et al., 2017). A dynamic balance between both types of pathways is supported by two pieces of evidence: (1) the simultaneous potentiation of BLA projections to non-sensitive OT neurons in the CeL and (2) the specific potentiation to CeL-sensitive OT neurons in those individuals that successfully acquire escape responses to imminent threat. In this process, the BLA can rapidly choose to activate either pathway, depending on threat imminence, which leads in HE rats to efficient escape behavior.

As noted, the neuroimaging research here from BLA-damaged humans and healthy controls suggested that escape opportunities from imminent threat crucially depend on BLA inhibition of the brainstem-freezing response via the CeA. Our finding that, after learning to escape from imminent threat, rats also show potentiation of different LA projections to the CeL is in line with this hypothesis and also with recent theories on distinct salience- and valence-selective neuronal networks within the BLA (Janak and Tye, 2015). Interesting in this respect is that our rat version of the TET provides only one escape pathway, namely to the adjacent compartment. As such, the increasing threat not only makes the employed designated compartment aversive but simultaneously renders the adjacent compartment rewarding. It is therefore also possible that valence-specific projections from the BLA to the nucleus accumbens (NAc) play a role in the final execution of an active, goal-directed response to the threat. The recent work of Namburi et al. (2015) suggests that there are two neuron populations in the BLA whose projections to the NAc and CeM underlie, respectively, increases in instrumental responding and increases in passive fear defensive responses. Competition between these two populations may therefore play a role in tipping the choice between freezing and escape behaviors. LeDoux et al. (2017) recently argued that a shuttling design, as we used here, trains the animal to express habitual, instrumental, escape behavior and that this habit formation is most likely subserved by this BLA-NAc pathway.

The present results suggest that this habitual escape behavior can be expressed independently from the amygdala in the case of distant threat, but when the escape action needs to be rapid (imminent threat), it can only be expressed efficiently when passive defensive responses are actively reduced through BLA-CeL interactions or pharmacological activation (TGOT) of the CeL. This insight not only adds to the increasing evidence for the role of the rodent (Balleine and Killcross, 2006, Phillips et al., 2003) and human (van Honk et al., 2013) BLA in instrumental, goal-directed economic behaviors but also extends this function of the BLA to the domains of threat processing during rapid escape and passive defensive behaviors.

Finally, our cross-species model of amygdala sub-region functioning also clarifies opposing behavioral observations on fear behaviors in UWD. In particular, the observation that UWD subject SM-046 has reduced fear experience (Feinstein et al., 2011) may seem to contradict the results presented here but can be explained by the fact that, in SM-046, both the BLA and the CMA are completely calcified. Consequently, the processing of new information by the amygdala is wholly lacking in this UWD subject so resulting in an absence of fear experience based on novel sensory information. In conclusion, to date, only a subset of the many rodent amygdala studies of defensive behavior has been successfully translated to humans (Janak and Tye, 2015). Moreover, none of these translations directly address the level of amygdala sub-regions. Here, we find, in spite of the relatively significant increase of size of the human BLA over the CeA as compared to rodents, a robust conservation of function between rodents and humans in the BLA. We started out with behavioral and neuroimaging observations in BLA-damaged humans, which suggested a key role for the BLA, in concert with the CeA, in the inhibition of passive defensive behaviors. Next, using a reversed translation of these human data to a rodent model, we were not only able to validate the human behavioral findings but also to uncover the pivotal underlying neurobiological mechanism. This mechanism, and specifically the pharmacological activation method employed, opens up new opportunities for the study and potential treatment of fear and anxiety disorders. That is, using our cross-species validated TET paradigm, we were able to pharmacologically restore deficient escape behavior by administering an oxytocin-receptor agonist directly in the rat CeL. Further research in human fear and anxiety using this TET paradigm (cf. Heesink et al., 2017) combined with pharmacological manipulation, perhaps using selective oxytocin agonists that are under development, is thus warranted. Notably, safety or escape behaviors are maladaptive in many of disorders of fear and anxiety, and oxytocin receptors may well provide a treatment target. In sum, our findings hold both fundamental and clinical relevance, but for deeper clinical-applicable insights into the role of this mechanism in pathological fear and anxiety, further biobehavioral research across humans and rodents is of the essence.

## STAR★Methods

### Key Resources Table

**Table:** 

REAGENT or RESOURCE	SOURCE	IDENTIFIER
**Antibodies**		

DsRed Polyclonal Antibody	Clontech Laboratories	Cat#632496; RRID: AB_10013483
Cy3-AffiniPure Goat Anti-Rabbit IgG (H+L) (min X Hu Sr Prot) antibody	Jackson ImmunoResearch Labs	Cat#111-165-045; RRID: AB_2338003

**Bacterial and Virus Strains**		

AAV_8_-CaMKIIα-hM4D-mCherry	University of North Carolina (UNC) Vector Core	Addgene AAV8; 50477-AAV8

**Chemicals, Peptides, Electrodes and Recombinant Proteins**		

Muscimol-Bodipy-TMR conjugated	ThermoFisher Scientifics	Cat#M23400; CAS: 2763-96-4
Oxytocin receptor agonist [Thr4,Gly7]-oxytocin (TGOT)	Bachem	Cat#H-7710.005; CAS: 60786-59-6
Oxytocin-receptor antagonist (CH2)5-Tyr(Me)-[Orn8]-vasotocin (OTA)	Bachem	Cat#H-2908.001; CAS: 114056-26-7
Clozapine n-oxide (CNO)	Tocris	Cat#6329; CAS: 34233-69-7
Picrotoxin	Sigma	Cat#P1675; CAS: 124-87-8
1,2,3,4-tetrahydro-6-nitro- 2,3-dioxo-benzo[f]quinoxaline-7-sulfonamide (NBQX)	Sigma	Cat#N171; CAS: 23357-52-0
Tetrodotoxin (TTX)	Sigma	Cat#4368-28-9; CAS: 4368-28-9
Glycin	Caelo	Cat#17026502; CAS: 56-40-6
DL-2-Amino-5-phosphonopentanoic acid (AP-5)	Sigma	Cat#A5282. CAS Number 76326-31-3
VECTASHIELD Antifade Mounting Medium with DAPI	Vector Laboratories	Cat#H-1200
Platinum-iridium parallel bipolar electrode	FHC	Cat#30211

**Deposited Data**		

FMRI: non-thresholded statistical maps	NeuroVault data repository (Gorgolewski et al., 2015)	https://neurovault.org/collections/KEFBYYQG/

**Experimental Models: Organisms/Strains**		

Rat: Sprague-Dawley OFA; Crl:OFA(SD)	Charles River	Cat#2312474; RRID:RGD_2312474

**Software and Algorithms**		

Med PC-IV	Med Associates, St. Albans, VT, USA	Cat#MED-SOF-735
Prism 7	GraphPad Software	https://www.graphpad.com/scientific-software/prism/
pClamp 10.2.	Axon Instruments	https://www.moleculardevices.com/
Clampfit 10.2	Axon Instruments	https://www.moleculardevices.com/
MiniAnalysis 6.0	Synaptosoft Inc	http://www.synaptosoft.com/MiniAnalysis/

**Other**		

Shuttle box Avoidance chamber	Med Associates, St. Albans, VT, USA	Cat#MED-APA-D1R
Acoustic Startle Reflex Package for Rat	Med Associates, St. Albans, VT, USA	Cat#MED-ASR-PRO1
Guide cannula 26 G	PlasticsOne	Cat#C315G/SPC
Micro-injector	PlasticsOne	Cat#C315I/SPC

### Contact for Reagent and Resource Sharing

Further information and requests for resources and reagents should be directed to and will be fulfilled by the Lead Contact, Ron Stoop (ron.stoop@unil.ch).

### Experimental Model and Subject Details

#### Human experiments

Five UWD subjects were tested in the ASR and fMRI experiments. The healthy control groups included fourteen (ASR experiment) and fifteen (fMRI experiment) matched (sex, age, socio-economic status and IQ) individuals. Five of the HC subjects participated in both experiments, and all participants were free from secondary psychopathology. See Table S1 for demographic information. UWD-sample-size was restricted to n = 5 due to UWD being extremely rare. The UWD subjects have participated in our previous behavioral (de Gelder et al., 2014, Klumpers et al., 2015, Morgan et al., 2012, Terburg et al., 2012, van Honk et al., 2013) and neuroimaging (Hortensius et al., 2016, Hortensius et al., 2017) work in this group, and this sample size has proven to be sufficient for identifying hypothesis-driven differences. Participants were unaware of the aim of the study and provided written informed consent. The study was approved by the Health Sciences Faculty Human Research Ethics Committee of the University of Cape Town and carried out in accordance with the standards set by the Declaration of Helsinki. Pre-processing of data was performed blinded from subject groups.

#### Animal experiments

We used Sprague-Dawley OFA male rats (Oncins France Strain A, Charles Rivers), 6-8 weeks old at the beginning of the experiment, weighing approximately 300 g. Rats were pathogen free and housed individually on a 12-h light/dark cycle (light on at 08:00) with *ad libitum* access to food and water *ad libitum*. Rats were handled for 5 min for a minimum of 3 d before behavioral experiment began. We used a new naive cohort of rats for each experiment. All procedures were approved by the veterinary office of the Canton of Vaud in Switzerland.

### Method Details

#### Human experiments

##### Threat and Escape Task

The TET was programmed in E-Prime 2.0 (Psychology Software Tools: https://pstnet.com/welcome-to-e-prime-2-0/). A detailed overview of the events in the TET is depicted in Figure S1. Each trial in the TET commenced with a rest phase consisting of a black fixation-cross on a white background. Next, in the anticipation phase, an image appeared that either represented the THREAT (a yellow shock pictogram) or SAFE (a blue no-shock pictogram) condition. These pictures appeared either full-screen (inescapable condition; IE), full-screen divided by 2 (imminent condition; I) or full-screen divided by 16 (escapable condition; E), and were presented for 4 to 9 s with an average of 6.5 for each condition.

Except for the SAFE/IE condition the pictures could ‘attack’ the participant. An attack in the THREAT/IE condition consisted of AS presentation. An attack in the E and I conditions consisted of a rapid increase in size of the pictures, which could be stopped by the participant with a button press (escape). When the participant failed to escape, and the pictures reached full-screen size, they were followed by AS presentation, but only in the THREAT condition. Immediately following this sequence of events, the next trial commenced.

In the ASR experiment startle sounds were presented during the anticipation phase in 9 out of 12 trials for each condition and were timed to occur 3 to 6 s after anticipation onset with an average of 4.5 s for each condition. Furthermore, to ensure startle habituation effects do not differentially affect task conditions the rest phase duration was adjusted trial by trial in such a way that the startle sounds were always 17, 20 or 23 s apart with an average of 20 s for each condition. In this algorithm AS presentation was startling in itself and thus also treated as startle stimulus.

Attacks occurred in 3 out of the 12 (ASR) or 15 (fMRI) trials in each condition. To ensure an even threat-level throughout the task, trials were presented in quasi-random order and attack trials were evenly distributed over time. Furthermore, speed of the attacking pictures was adjusted individually to ensure that each participant could escape the THREAT/I condition at chance-level. To this end, participants engaged in an elaborate practice session preceding the experiment. Participants first performed 20 trials of a reaction time task (RTT) wherein they were instructed to press a button as fast as possible when a gray rectangle (similar in size to the E condition) started to grow in size. Reaction times were recorded and averaged. Next, after a thorough explanation of the TET the participants engaged in a 36-trial practice session with visual feedback instead of AS presentation, and a 100% attack rate. Average reaction time from the RTT was used as baseline duration of the imminent attacks (DIA). Whenever the participant failed to escape an imminent attack (button press too late) DIA was adjusted by adding 33ms (two frames at a display-refresh rate of 60Hz). Whenever the participant did successfully escape an imminent attack DIA was adjusted by subtracting 33ms. Duration of escapable attacks was always set at DIAx4.

Finally, in the ASR experiment participants engaged in a 12-trial practice session with a similar percentage of attacks (25%) as in the actual TET. This phase also included presentation of the startle sound in the anticipation phase of each trial and ASRs to these stimuli were used as habituation phase in data analysis. DIA was adjusted as before and the resulting DIA was used as baseline DIA in the actual experiment. During the experiment DIA-adjustment continued, but now based on threat trials only to ensure that reduced motivation to press the button during the safe condition would not result in slower attacks. Furthermore, in order to keep anticipation for motor responses in THREAT and SAFE conditions comparable, participants were explicitly instructed to also initiate escape during the SAFE trials.

The TET procedure was overall similar in both experiments with a few notable distinctions. In the fMRI experiment the startle stimuli were omitted and the AS consisted of a ∼110 dB(A) female scream. Accordingly, the visual stimuli used were ‘sound’ and ‘no sound’ pictograms. Furthermore, to increase power three trials were added to each condition except for the SAFE/IE trials. The latter was done as the attack trials are treated as nuisance regressors in our GLM models (see below) and this ensures that there is an equal amount (12) of trials for each condition in our analyses. Finally, due to the absence of startle sound stimuli, duration of the rest and anticipation phase could be shortened to 3-4 s (average 3.5 for each condition) and 3-6 s (average 4.5 for each condition) respectively.

##### Aversive stimuli

Aversive stimulus in the ASR experiment consisted of a shock to the wrist administered using tin cup electrodes on the left wrist, which were connected to a constant current stimulator (Digitimer DS7A, Digitimer Ltd). Preceding the experiment, the participants engaged in a shock work-up procedure which aimed to acquaint the participants with the shocks and to provide a uniform level of aversiveness across the participants. After electrode placement the work-up consisted of five sample shocks rated by the participants on a five-point scale ranging from ‘not annoying at all’ to ‘very annoying’. Shock levels were adjusted in order to achieve an intensity that was rated as four on this scale, which corresponded to ‘quite annoying’. For electrical stimulation, a train of 150 2-ms pulses was administered at a rate of 200 Hz.

To avoid fMRI signal deterioration due to the presence of shock-electrodes in the MRI-chamber we used for the fMRI experiment an AS consisting of a 1 s, ∼110 dB(A), female scream presented through MRI-compatible headphones, which has been shown to be comparable to the electric shock in eliciting threat potentiation of ASR (Glenn et al., 2012). After the fMRI-experiment the participants were asked to rate their fear for AS presentation on a 9-point scale ranging from ‘not fearful at all’ (1) to ‘fearful’ (5) to ‘very fearful’ (9). There was no group-difference in these ratings (p = 0.78). HCs had an average rating of 6.1 (SEM = 0.3) and UWDs an average of 6.2 (SEM = 0.4), which for both groups translates to a rating between fearful and very fearful.

##### Acoustic Startle Response measurements

Startle reflexes were probed by presenting ∼107 dB(A) bursts of 50 ms white noise with near instantaneous rise time. Electromyographic recording of the startle reflex was carried out using the Biosemi Active Two system (http://www.biosemi.nl) with Ag-AgCL electrodes positioned over the orbicularis oculi muscle below the right eye. One electrode was located below the pupil and the other ∼15 mm toward the lateral canthus of the eye.

##### Reaction time measurement

Reaction times were recorded throughout the ASR and fMRI experiments. Speed of the attacks in both experiments adjusted to performance of the participant in such a way that imminent trials could be escaped from in half of the trials. In line with this no significant differences were observed between HCs and UWDs on average number of aversive stimulus presentations (ASR experiment: HCs = 4.4, UWDs = 4.8, p = 0.31; fMRI experiment: HC = 4.4, UWD = 4.2, p = 0.47). Average reaction time differences were also non-significant (ASR experiment: HCs = 351 ms, UWDs = 427 ms, p = 0.09; fMRI experiment: HCs = 339 ms, UWDs = 361 ms, p = 0.51)

##### Magnetic resonance imaging sequences

MRI-scans were acquired with a Siemens Magnetom Allegra 3-Tesla head-only scanner at the Cape Universities Brain Imaging Centre (CUBIC) in Cape Town, South Africa. For lesion analysis, we obtained whole brain T2-weighted images with 1mm isotropic resolution, TR = 3500 ms, and TE = 354 ms. Functional whole brain MRI-scans were obtained with a 2D-EPI sequence with 36 slices in interleaved-ascending order, 3.5 mm isotropic resolution, Flip-angle = 70°, TR = 2000 ms, TE = 27 ms, and EPI-factor = 64. The first 4 volumes were acquired prior to the start of the fMRI task, and discarded from analyses. For normalization of functional scans, we obtained structural T1-weighted images with 1mm isotropic resolution, TR = 2300 ms, and TE = 393 ms.

#### Animal experiments

##### Behavior: Threat and Escape Task

Rats were trained in a modified threat-conditioning paradigm using a “two-way shuttle box” (Med Associates, St. Albans, VT, USA) wherein threat and escape possibilities were dynamically changed. The shuttle box was separated into two parts by a door that allowed movement from one compartment to the other. Rats were conditioned to repeatedly experience the “circa-strike” phase (contact with predator is occurring or inevitable) of a virtual predator attack (Fanselow, 1994). This was achieved through presentation of tones (conditioned stimulus, CS) that differed in frequency, intensity and length, and culminate in the presentation of an electric shock (unconditioned stimulus, US; 0.5 mA, 5 s maximum) at the end of the tone. The animals learned to escape by moving to the other compartment during the presentation of the tone. Failing to escape resulted in presentation of the US, which lasted 5 s, unless the animal moved to the other compartment during the shock. Rats were trained to different tones of decreasing duration, but increasing frequency (4 kHz/15 s, 8 kHz/10 s, 10 kHz/5 s, 12 kHz/1 s), with higher frequency announcing higher imminence of the threat.

After habituation in the shuttle box for 45 minutes on the first day, rats were trained on three consecutive daily sessions with 4 kHz/15 s and 8 kHz/10 s tones. After three sessions, we selected animals who reached at least 60% of successful escape responses and on the next days we tested the 10 kHz/5 s and 12 kHz/1 s tones. On the following day, rats received intraperitoneal Veh or CNO and after 30 minutes were tested again following the same procedure. For the experiments with TGOT and OTA, rats received intracerebral microinjection with TGOT or OTA or equivalent volume of Vehicle 15 minutes before the beginning of the session. For the combined chemogenetic and pharmacological experiment: rats received intraperitoneal Veh or CNO 30 minutes before the beginning of the session. After 15 minutes they were injected also with Vehicle or TGOT intracerebrally before the experiment began. Freezing in the inescapable threat condition has been measured in a different set of experiments, due to appearance of learned helplessness behavior that can affect escape behavior on the other conditions. For inescapable threats conditioning, rats were placed in the shuttle box using the same setting of the TET and were presented with 1 s tone (eight times) always culminating in an electric shock. In this condition, shuttling to the adjacent compartment did not terminate the tone and did not give chance to avoid the shock. Of note: Although seemingly similar, the inescapable threat is essentially different from traditional fear conditioning in which, during testing, the animal is not anymore exposed to the shock, but only to the tone.

Only rats that were not able to learn the task (TET) were excluded (n = 5). All behavioral experiments were replicated three to four times. Rats’ behaviors were measured by freezing, escape responses and latency to escape (in seconds).Freezing behavior was expressed as percentage of time freezing over a period of 60 s after the end of CSs. For freezing behavior, we considered the total absence of all movements, excepted for those related to respiration, lasting for at least one second. Freezing was manually analyzed by post hoc observation of video recordings by an experimenter blind to the treatments. Escape responses and latency to escape were measured by a software (Med PC-IV, Med Associates, St. Albans, VT, USA). Escapes were expressed as percentage of successful anticipation of the shock during presentation of the tone and finally assigned to the animals by an experimenter blind to the treatments.

##### Behavior: Acoustic startle response

We tested rats in a ventilated acoustic startle reflex chamber with a clear Plexiglas cylinder (Med Associates, St. Albans, VT, USA). The startle paradigm used in this study follows a basic design consisting of 1 day of startle acclimation, 2 day of startle baseline, 1 day of conditioning recall (TET), followed by testing of the potentiation of the startle response. For assessment of startle baseline, rats were exposed to 2 sessions of 30 presentations of a 50ms white noise burst startle stimulus at 95, 105, or 115dB (10 of each) given in a predetermined pseudo-random order with a 15 s inter-trial interval. These sessions served for acclimating the subjects to the experimental environment and permitting the calculation of the mean ‘pre-threat’ startle baseline to be constructed for each subject. The baseline was the mean of the startle amplitudes of all the trials over the 2 days. On the fourth day, all rats were tested in the TET. On the next day we measured the potentiation of the startle reaction. Rats received intraperitoneal Veh or CNO 30 minutes before the beginning of the startle potentiation testing. For the experiment with TGOT, rats received intracerebral Veh or TGOT 15 minutes after intraperitoneal injection of Veh or CNO. After 15 minutes of recovery in the home-cage, they were tested for startle potentiation. All rats were drug naive before this experiment. This test consisted of a 5min acclimation period followed by 70 startle trials with 15 s intervals. The first 10 trials consisted of 95, 105, or 115dB noise bursts presented in a predetermined pseudo-random order to re-acclimate subjects to the startle stimuli. The next 60 trials consisted of 95, 105, or 115 dB noise bursts co-terminating with the 4 kHz/15 s tone or the 12 kHz/1 s, previously used in the TET. For each noise burst intensity, 10 trials were presented in the presence of the 4 kHz/15 s-tone and 10 trials were presented in the presence of the 12 kHz/1 s-tone. Potentiation of the startle response was calculated as percentage of the habituation and values were assigned to the animals by an experimenter blind to the treatments.

##### Virus construct

AAV_8_-CaMKIIα-hM4D-mCherry virus (3.3x10e12 virus mol/mL) was produced at the Gene Therapy Center Vector Core in the University of North Carolina at Chapel Hill. The vector is a standard hM4D(Gi) receptor constructed under the control of CaMKIIa, with an mCherry reporter, for CNO-induced neuronal silencing. This vector is now available at Addgene: 50477-AAV8. Behavioral experiments (TET or ASR) were performed 4 weeks after virus injection. For chemogenetic silencing of the BLA, all rats were injected with AAV_8_-CaMKIIα-hM4D-mCherry randomly divided into Vehicle or CNO groups. At the end of the experiment rats were sacrificed and their brains were sectioned to check the expression of the virus.

##### Animal surgery

For virus injections, 6-8 weeks old rats were deeply anesthetized with 5% isoflurane (vol/vol) in oxygen and kept at 1.5% isoflurane during surgery. Surgery was performed with a stereotaxic frame (Kopf). Coordinates (in mm) for viral injection in the BLA: AP: −2.8; ML ± 5.2; DV 7.6 – 8 – 8.4. All infusions were performed using a 28 gauge Hamilton syringe connected to an infusion pump (0.15 ul/min). Each rat received 0.9 ul of AAV construct at each site.

For behavioral experiments that required drug infusion, guide cannulae cut 5.8 mm below the pedestal (26 G, PlasticsOne) were bilaterally implanted for direct intra-amygdala injections. For this purpose, animals were deeply anesthetized with 5% isoflurane in pure oxygen and their heads were fixed in a stereotaxic fame. The skull was exposed and two holes were drilled according to coordinates that were adapted from a rat brain atlas to target the CeA (AP: −2.3 mm; ML ± 4 mm; DV −7.5 mm relative to bregma) by comparing the typical bregma-lambda distance (9 mm) with the one measured in the experimental animal. Two screws were fixed to the caudal part of the skull in order to have an anchor point for the dental cement. The acrylic dental cement was finally used to fix the cannulae and the skin was sutured. Cannula positions were verified post hoc by injection of fluorescently labeled muscimol (Muscimol-Bodipy-TMR conjugated) at a concentration of 1.6 mM as previously showed (Viviani et al., 2011).

##### Ex vivo electrophysiology

About 30 min after behavioral session end, rats were deeply anaesthetized with 5% isoflurane, and the brains were rapidly removed. Coronal sections containing the LA and CeL (350 μm) were cut using a vibratome (Compresstome VF-200, Precisionary Instruments, Greenville, NC, USA) in ice-cold NMDG solution, saturated with 95% O_2_ and 5% CO_2_, containing (in mM): 110 NMDG, 110 HCl, 25 NaHCO3, 25 glucose, 3 KCl, 1.1 NaH2P04^∗^H20, 0.5 CaCl2^∗^2H20, 10 MgCl2^∗^6H20, 10 ascorbic acid, 3 pyruvic acid (pH was adjusted to 7.3 with NaOH and osmolarity was 300 mOsm). Brain slices were incubated at 32–34°C in the same solution, to recover for 20 min before the beginning of the experiment. Recordings were made from visually identified neurons in the CeL amygdala using infrared differential interference contrast optics (Leica DM LFS). Whole cell recordings were performed using borosilicate glass electrodes (4-6 MΩ, Sutter Instruments, CA, USA), filled with (in mM): 135 caesium methansulphonate, 10 HEPES, 1 EGTA, 10 tetraethylammonium chloride, 4 MgATP, 0.3 NaGTP (pH 7.25–7.4 adjusted with CsOH; 280–290 mOsm). Whole cell recordings were acquired with Axopatch 200B (Molecular Devices, Sunnyvale, CA, USA), sampled with a Digidata 1440A interface, filtered at 2 kHz and digitized at 10 kHz with pClamp 10.2. Series resistance (10–20 MΩ) and input resistance were continuously monitored throughout the experiment.

##### Evoked excitatory postsynaptic currents

AMPA and NMDA EPSCs were recorded in artificial cerebrospinal fluid containing in mM: 125 NaCl, 25 NaHCO3, 4 KCl, 1.2 NaH2PO4, 2 MgCl2, 0.5 CaCl, 10 D-Glucose), with 50 μM picrotoxin and 50 μM glycine. For the electrical stimulation a platinum-iridium parallel bipolar electrode (FHC, Bowdoin, ME USA) was placed in the LA in close proximity to the recording pipette located in the CeL (Figure 5). Stimuli with duration of 100 μsec were delivered in pairs, with an inter-stimulus interval of 60 ms. The stimulation intensity was adjusted in order to obtain an evoked EPSC with an amplitude of 100 pA, with a short and constant latency (2–3 ms). Evoked EPSCs were recorded at –70 mV (for AMPA currents) and +40 mV (for NMDA currents). EPSCs were evoked every 10 s (0.1 Hz).

##### Functional assessment of oxytocin receptor expression in CeL

The expression of OTR in the CeL was assessed pharmacologically and electrophysiologically, by using the highly specific OTR agonist TGOT (Thr^4^,Gly^7^]OT peptide). For each neuron, after measuring the AMPA/NMDA ratio, the firing threshold was measured in current clamp using a protocol consisting of 2 ms incremental current injections in steps of 10 pA. The threshold was considered as the value of the current injection at which the first action potential occurred. After measuring the firing threshold in baseline conditions, 1 μM TGOT was applied in the bath for 3 minutes and the threshold was assessed again during the TGOT treatment and up to 20 minutes after (once every 30 to 60 s). The cells that showed at least a 10% decrease in the threshold current (the excitability was increased by TGOT) were identified as OTR+ (Huber et al., 2005).

In a control experiment, the specific oxytocin receptor antagonist (OTA) was applied before TGOT, in the same protocol of current injections to assess any change caused by the blockade of these receptors in CeA, which might indicate differences in signaling levels of endogenously released oxytocin. In this experiment, 1 μM OTA was applied in the bath for 3 minutes and the AP threshold was assessed again during the OTA treatment (once every 30 to 60 s). Posteriorly, TGOT was applied to identify whether those cells expressed the oxytocin receptor.

##### AMPA/NMDA ratio analysis

Fifteen traces recorded at either −70 mV or +40 mV were averaged, using Clampfit 10.2 software. Traces containing stimulus-evoked epileptiform events (reflecting inhibitory blockade with 50 μM picrotoxin) were excluded from the average waveform. The AMPA component was measured as the peak of the first evoked current recorded at −70 mV, and the NMDA component was measured as the average current during a 10 ms interval, 40 ms after the onset of the stimulation (for the first stimulation in a pair). The AMPA/NMDA ratio was calculated as *I*_AMPA_ at −70 mV / *I*_*NMDA*_ at +40 mV. In order to confirm the identity of the currents and to establish the time points for analysis for each components, control experiments using 10 μM NBQX (an AMPA/kainate receptors antagonist) and 50 μM AP-5 (an NMDA antagonist) at the two holding potentials were performed.

##### Spontaneous quantal excitatory activity onto different populations in CeA

Spontaneous mEPSCs were recorded in the presence of TTX (1 μM). Spontaneous synaptic events were captured continuously for 5 minutes in already identified oxytocin sensitive and non-sensitive populations, from the different behavioral groups. All events were detected offline and their amplitude and frequency calculated using MiniAnalysis 6.0 (Synaptosoft). A change of mEPSCs amplitude is consistent with an increase in AMPA receptor density in postsynaptic structures that already possess the receptor. A change in mEPSC frequency is consistent with either a presynaptic change in release probability or the insertion of postsynaptic AMPA receptors y that increases detection sensitivy for previously undetected mEPSCs. As a control experiment, 10 μM NBQX was applied at the end of the recording which inhibited all mEPSCs. This showed that they were AMPA-R dependent currents.

##### Chemicals

Rats received bilateral injections of 0.5 μl containing vehicle (artificial cerebrospinal fluid, ACSF), specific oxytocin receptor agonist [Thr4,Gly7]-oxytocin (TGOT, 7 ng; Bachem) or oxytocin-receptor antagonist (CH2)5-Tyr(Me)-[Orn8]-vasotocin (OTA, 42 ng; Bachem) dissolved in ACSF. For this procedure two injectors (cut to fit 5.8 mm guide cannulae protruding 2 to 2.5 mm beyond the lower end of the cannulae) were bilaterally lowered into the guide cannulae, connected via polyten tubing to two Hamilton syringes that were placed in an infusion pump and 0.5 μl of liquid was injected in each hemisphere over a period of 2 minutes. Then the injectors were kept in place for an additional minute in order to allow a complete diffusion of liquid throughout the tissue. Rats were subsequently left in the home cage for 15 minutes to recover from the stress of the injection and then placed in the testing chamber.

Clozapine n-oxide (CNO, Tocris) was dissolved in sterile water and administered, 30 minutes before testing, by intraperitoneal injection in a volume of 1 ml/kg at doses of 3 mg/kg. Previous study has found that rats expressing DREADD receptors and treated with vehicle do not differ from those expressing the GFP control transgene and treated with CNO (Ferguson et al., 2011). Therefore, only vehicle-treated DREADD receptor groups were used as controls in the present experiments.

##### Immunofluorescence

To verify that viral injection and expression were localized in the target region, rats were deeply anaesthetized using sodium pentobarbital and intracardially perfused with PBS followed by 4% buffered paraformaldehyde (PFA). Brains were removed, post-fixed for 1 hour in 4% PFA and cryoprotected in 30% sucrose. 50-μm coronal brain slices were cut with a freezing microtome (Microm, Thermo Fisher Scientific, Waltham, MA, USA) and stored in PBS. Standard immunohistochemical procedures were performed on free-floating brain sections. Sections were incubated for 3 hours at RT in blocking solution (1% normal goat serum, 2.5% bovine serum albumin, 0.3% Triton X-100 in PBS), followed by overnight incubation with primary antibody (rabbit anti-dsRed, Clontech, 632496) in blocking solution at 4°C on shaker. The next day, slices were washed 3 times in phosphate-buffered saline + 0.3% Triton X-100 (PBS-T), and then incubated with secondary antibodies (Cy3-tagged goat anti rabbit IgG, Jackson IR, 111-165-045) in PBS-T for 2 h at room temperature. After the secondary antibody incubation, slices were washed 3 times, stained with 4,6-diamidino-2-phenylindole 1:10 000 coupled with the mounting medium antifade Vectashield medium (Vector Laboratories, Burlingame, CA, USA). Slices were mounted on gelatin coated glass slides, coverslipped and imaged using an Axioscan Z1 (Carl Zeiss AG).

### Quantification and Statistical Analysis

All statistical details can be found in the figures, figure legends and supplemental tables.

#### Human experiments

##### ASR analysis

Startle data were preprocessed in Brain Vision Analyzer 2 (Brain Products GmbH: https://www.brainproducts.com/downloads.php?kid=9) according to previously published guidelines (Blumenthal et al., 2005). In brief, startle data were segmented, bandpass filtered (28–500 Hz, 24 dB/oct), rectified, smoothed and baseline (50 ms before stimulus onset) corrected. The highest peak in the resulting signal was taken as the amplitude of the response. Consistent with previous work (Klumpers et al., 2010) data were checked for artifacts such as spontaneous blinks and movement in the analysis window. Trials with excessive activity in the 50 ms baseline period immediately preceding the response (exceeding the mean baseline activity for that subject by more than 2 *SD*s) were scored as missing values. Also, trials with peak amplitude latencies outside the normal range (25-115 ms post startle probe) were set to missing. Following this procedure resulted in artifact-rejection of 8.8% of the trials.

ASRs from the experimental phase were divided by the mean ASRs from the habituation phase in order to control for overall ASR differences due to external factors. The resulting ASR-proportions were analyzed using linear generalized estimating equations (GEE) models with a model based covariance matrix and an autoregressive working correlation matrix based on trial order. These models are robust to non-normality and missing data due to artifact-rejection and sensitive for trial by trial individual differences while controlling for trial by trial dependencies.

##### Lesion analysis

To estimate extent and anatomical location of the lesions T2-weighted scans were normalized to MNI-space using unified segmentation, which is optimized for normalization of lesioned brains (Crinion et al., 2007). Lesion volumes were defined using the 3D volume-of-interest featured implemented in MRIcroN (https://www.mccauslandcenter.sc.edu/mricro/mricron/index.html). Based on MR-images the precise borders between amygdalae and neighboring structures, or between the sub-regions of the amygdala, cannot be established (Amunts et al., 2005, Solano-Castiella et al., 2010). To determine the precise location of the lesions in our UWD subjects, we therefore assigned the lesion volumes to cytoarchitectonic probability maps according to the method described by Eickhoff et al., (2007). In this method, that is implemented in the SPM8 anatomy toolbox (http://www.fz-juelich.de/inm/inm-1/DE/Forschung/_docs/SPMAnatomyToolbox/SPMAnatomyToolbox_node.html), a volume of interest is superimposed onto a cytoarchitectonic probability map of the medial-temporal lobe (Amunts et al., 2005). This map is based on microscopic analyses of ten postmortem human brains and follows a generally accepted division of the human amygdala in three sub-regions. The first is the central-medial amygdala (CMA), which consists of the central and medial nuclei. The second is the basolateral amygdala (BLA), which includes the lateral, basolateral, basomedial, and paralaminar nuclei, and the third is the superficial (or corticoid) amygdala (SFA), which includes the anterior amygdaloid area, amygdalopyrifom transition area, amygdaloid-hippocampal area, and the cortical nucleus (Amunts et al., 2005). This method assigns to any given voxel a value representing the probability that it belongs to an underlying structure. These are derived from an overlap analysis of ten postmortem brains, and are therefore divided in ten separate probability classes ranging from 10% to 100% probability.

To estimate how well the lesion volumes fit to the underlying structure, *P*_excess_ values are computed using the following equation:

$$P_{\mathrm{excess}}=P_{\mathrm{lesion}}/P_{\mathrm{map}}$$

Whereby *P*_*lesion*_ represents the average cytoarchitectonic probability of the voxels that are shared by the lesion and the cytoarchitectonic probability map, and *P*_*map*_ represents the average probability of the whole structure’s cytoarchitectonic map. These values thus represent how much the average probability of the overlapping voxels exceed the overall probability distribution of that particular structure, and thus indicate whether the lesion overlaps with relatively high or low probability classes of that structure. In other words, *P*_*excess*_ represents how ‘central’ the location of the lesion is relative to that structure’s cytoarchitectonic map, whereby *P*_*excess*_ > 1 indicates a more central, and *P*_*excess*_ < 1 a more peripheral location (Eickhoff et al., 2007).

##### fMRI analysis

Preprocessing and subsequent analyses were performed with SPM8 (https://www.fil.ion.ucl.ac.uk/spm), and followed the procedures from previous research with the TET (Montoya et al., 2015). Functional scans of both sessions were motion corrected to the first dynamic scan and slice-time corrected to the middle slice. The T1-weighted scan was then coregistered to the mean functional scan. Subsequently, using unified segmentation, the structural scan was segmented and normalization parameters were estimated. Using these normalization parameters, all volumes were normalized to a standard brain template (MNI) and resliced at 2.0 mm isotropic voxel size. Smoothing with an 8.0 mm full width at half maximum Gaussian kernel was applied to the normalized functional volumes.

Brain activity related to threat anticipation were investigated within general linear models (GLM). The TET was designed to measure BOLD-responses during the anticipation phase of passive fear for, or active escape from, an aversive stimulus. Therefore, trials without actual attacks (12 trials for each condition) were of main interest, whereas trials with attacks (3 trials for each condition excluding safe/inescapable) were treated as separate variables in the model, which ensures that the effects of motion related artifacts due to button-presses and AS presentation do not affect our measure of interest. Thereto, in the first-level GLM for each test-session, we used twelve regressors for our trials of interest: Six for the trial-onsets (box-car function for stimulus-duration, 3-6 s), and six for the trial-offsets (delta function). Trial-offset regressors were included based on a previous study into threat-offset effects (Klumpers et al., 2010) but considered of no-interest for the current study. Furthermore, ten other nuisance regressors were defined: Five for the trial-onsets for stimuli that actually attacked, four for the attack-onset (box-car function for attack-duration), and one for the AS-onset (box-car function for AS-duration, 1 s).

These regressors were all convolved with the hemodynamic response function as implemented in the SPM8 software. In addition, realignment parameters and a discrete cosine transform high-pass filter with a 1/128 Hz cut-off frequency were entered into the analyses to reduce variance due to nuisance factors such as movement and drifts in the signal. Thus, in total twenty-nine regressors were entered in the first level statistical analysis. For each subject, and session, we computed contrast maps for onset of distant, imminent and inescapable threat and safe cues versus baseline.

For the second level analysis, the contrast maps of safe and threat onset from both groups were entered in a full-factorial 2X2X3 ANOVA design with GROUP (UWDs and HCs) as between-subjects factor and CONDITION (threat and safe) and DISTANCE (distant, imminent, inescapable) as within-subject factors.

All calculated linear contrasts were thresholded at p < 0.005, and the resulting clusters were family-wise error (FWE) corrected with a threshold of p < 0.05. To link activation patterns to anatomy, the significant clusters were inspected with the automated anatomical AAL template (Tzourio-Mazoyer et al., 2002). For regions of interest (ROIs), we applied small volume corrections and for analysis consistency we report these results with the same statistical thresholds. Importantly, applying a more conservative voxel-based FWE correction (p < 0.05) for these smaller volumes did not change the results. ROIs included bilateral masks for the amygdala based on the cytoarchitectonic probability maps (Amunts et al., 2005) implemented in the anatomy toolbox for SPM8 (Eickhoff et al., 2005) and the midbrain and pons as included in the AAL atlas implemented in SPM8 (Tzourio-Mazoyer et al., 2002).

Finally, we performed a functional connectivity analysis using the psycho-physiological interaction function in SPM8 to further investigate the group differences in brain activation. For each participant, the time-course (first-eigenvariate) was extracted from a 6 mm sphere around the peak voxel identified in the group-level three-way interaction in the pons (MNI = 6, −26, −23), and mean-corrected for general task effects. An interaction term was computed with the threat > safe contrast, and both were entered in a new GLM with all task regressors. Contrast maps for the interaction term were computed and entered in a second-level GLM to test for group differences in threat-related pons connectivity. In order to maintain power, we limited this analysis to the identification of connectivity differences in the overall CONDITION (threat versus safe) contrast. Although this approach does not allow to test for connectivity modulations based on specific threat distances, it does provide insight into connectivity pattern modulations across dynamically changing threatening compared to safe conditions in general.

#### Animal experiments

##### Statistics

Statistical parameters including the exact value of n, precision measures (mean ± SEM) and statistical significance are reported in the Figure Legends. All data from animal behavior were statistically analyzed using Prism 7 (GraphPad Software). To evaluate the normality of our data we pooled data from TET test conducted under naive conditions and vehicle treatments and we used D’Agostino and Pearson normality test. For the analysis of variance, we used ANOVA. *Post hoc* analyses were conducted using Bonferroni test with multiple comparisons corrections, when statistical significance emerged in the main effects or interactions. The accepted value for significance was p < 0.05. All data are shown as mean and s.e.m. No statistical methods were used to predetermine sample size. Result sheets of statistical tests detailing (wherever applicable) estimates of variance within each group, confidence intervals and comparison of variances across groups are available upon request.

### Data and Software Availability

#### Human experiments

Non-thresholded statistical maps of the main fMRI analyses can be found at the NeuroVault data repository (Gorgolewski et al., 2015): https://neurovault.org/collections/KEFBYYQG/. Startle data are available on request from the authors.

## References

[bib1] Alexander G.M., Rogan S.C., Abbas A.I., Armbruster B.N., Pei Y., Allen J.A., Nonneman R.J., Hartmann J., Moy S.S., Nicolelis M.A. (2009). Remote control of neuronal activity in transgenic mice expressing evolved G protein-coupled receptors. Neuron.

[bib2] Amunts K., Kedo O., Kindler M., Pieperhoff P., Mohlberg H., Shah N.J., Habel U., Schneider F., Zilles K. (2005). Cytoarchitectonic mapping of the human amygdala, hippocampal region and entorhinal cortex: intersubject variability and probability maps. Anat. Embryol. (Berl.).

[bib3] Balleine B.W., Killcross S. (2006). Parallel incentive processing: an integrated view of amygdala function. Trends Neurosci..

[bib4] Blumenthal T.D., Cuthbert B.N., Filion D.L., Hackley S., Lipp O.V., van Boxtel A. (2005). Committee report: Guidelines for human startle eyeblink electromyographic studies. Psychophysiology.

[bib5] Ciocchi S., Herry C., Grenier F., Wolff S.B., Letzkus J.J., Vlachos I., Ehrlich I., Sprengel R., Deisseroth K., Stadler M.B. (2010). Encoding of conditioned fear in central amygdala inhibitory circuits. Nature.

[bib6] Crinion J., Ashburner J., Leff A., Brett M., Price C., Friston K. (2007). Spatial normalization of lesioned brains: performance evaluation and impact on fMRI analyses. Neuroimage.

[bib7] Davis M. (2006). Neural systems involved in fear and anxiety measured with fear-potentiated startle. Am. Psychol..

[bib8] Davis M., Whalen P.J. (2001). The amygdala: vigilance and emotion. Mol. Psychiatry.

[bib9] Davis M., Gendelman D.S., Tischler M.D., Gendelman P.M. (1982). A primary acoustic startle circuit: lesion and stimulation studies. J. Neurosci..

[bib10] de Gelder B., Terburg D., Morgan B., Hortensius R., Stein D.J., van Honk J. (2014). The role of human basolateral amygdala in ambiguous social threat perception. Cortex.

[bib11] Eickhoff S.B., Stephan K.E., Mohlberg H., Grefkes C., Fink G.R., Amunts K., Zilles K. (2005). A new SPM toolbox for combining probabilistic cytoarchitectonic maps and functional imaging data. Neuroimage.

[bib12] Eickhoff S.B., Paus T., Caspers S., Grosbras M.H., Evans A.C., Zilles K., Amunts K. (2007). Assignment of functional activations to probabilistic cytoarchitectonic areas revisited. Neuroimage.

[bib13] Fadok J.P., Krabbe S., Markovic M., Courtin J., Xu C., Massi L., Botta P., Bylund K., Müller C., Kovacevic A. (2017). A competitive inhibitory circuit for selection of active and passive fear responses. Nature.

[bib14] Fanselow M.S. (1994). Neural organization of the defensive behavior system responsible for fear. Psychon. Bull. Rev..

[bib15] Feinstein J.S., Adolphs R., Damasio A., Tranel D. (2011). The human amygdala and the induction and experience of fear. Curr. Biol..

[bib16] Ferguson S.M., Eskenazi D., Ishikawa M., Wanat M.J., Phillips P.E., Dong Y., Roth B.L., Neumaier J.F. (2011). Transient neuronal inhibition reveals opposing roles of indirect and direct pathways in sensitization. Nat. Neurosci..

[bib17] Glenn C.R., Lieberman L., Hajcak G. (2012). Comparing electric shock and a fearful screaming face as unconditioned stimuli for fear learning. Int. J. Psychophysiol..

[bib18] Gorgolewski K.J., Varoquaux G., Rivera G., Schwarz Y., Ghosh S.S., Maumet C., Sochat V.V., Nichols T.E., Poldrack R.A., Poline J.B. (2015). NeuroVault.org: a web-based repository for collecting and sharing unthresholded statistical maps of the human brain. Front. Neuroinform..

[bib19] Gozzi A., Jain A., Giovannelli A., Bertollini C., Crestan V., Schwarz A.J., Tsetsenis T., Ragozzino D., Gross C.T., Bifone A. (2010). A neural switch for active and passive fear. Neuron.

[bib20] Hamada T., McLean W.H., Ramsay M., Ashton G.H., Nanda A., Jenkins T., Edelstein I., South A.P., Bleck O., Wessagowit V. (2002). Lipoid proteinosis maps to 1q21 and is caused by mutations in the extracellular matrix protein 1 gene (ECM1). Hum. Mol. Genet..

[bib21] Haubensak W., Kunwar P.S., Cai H., Ciocchi S., Wall N.R., Ponnusamy R., Biag J., Dong H.W., Deisseroth K., Callaway E.M. (2010). Genetic dissection of an amygdala microcircuit that gates conditioned fear. Nature.

[bib22] Heesink L., Edward Gladwin T., Terburg D., van Honk J., Kleber R., Geuze E. (2017). Proximity alert! Distance related cuneus activation in military veterans with anger and aggression problems. Psychiatry Res. Neuroimaging.

[bib23] Hermans E.J., Henckens M.J., Roelofs K., Fernández G. (2013). Fear bradycardia and activation of the human periaqueductal grey. Neuroimage.

[bib24] Hortensius R., Terburg D., Morgan B., Stein D.J., van Honk J., de Gelder B. (2016). The role of the basolateral amygdala in the perception of faces in natural contexts. Philos. Trans. R. Soc. Lond. B Biol. Sci..

[bib25] Hortensius R., Terburg D., Morgan B., Stein D.J., van Honk J., de Gelder B. (2017). The basolateral amygdalae and frontotemporal network functions for threat perception. eNeuro.

[bib26] Huber D., Veinante P., Stoop R. (2005). Vasopressin and oxytocin excite distinct neuronal populations in the central amygdala. Science.

[bib27] Janak P.H., Tye K.M. (2015). From circuits to behaviour in the amygdala. Nature.

[bib28] Klumpers F., Raemaekers M.A., Ruigrok A.N., Hermans E.J., Kenemans J.L., Baas J.M. (2010). Prefrontal mechanisms of fear reduction after threat offset. Biol. Psychiatry.

[bib29] Klumpers F., Morgan B., Terburg D., Stein D.J., van Honk J. (2015). Impaired acquisition of classically conditioned fear-potentiated startle reflexes in humans with focal bilateral basolateral amygdala damage. Soc. Cogn. Affect. Neurosci..

[bib30] Knobloch H.S., Charlet A., Hoffmann L.C., Eliava M., Khrulev S., Cetin A.H., Osten P., Schwarz M.K., Seeburg P.H., Stoop R., Grinevich V. (2012). Evoked axonal oxytocin release in the central amygdala attenuates fear response. Neuron.

[bib31] LeDoux J.E., Moscarello J., Sears R., Campese V. (2017). The birth, death and resurrection of avoidance: a reconceptualization of a troubled paradigm. Mol. Psychiatry.

[bib32] Li H., Penzo M.A., Taniguchi H., Kopec C.D., Huang Z.J., Li B. (2013). Experience-dependent modification of a central amygdala fear circuit. Nat. Neurosci..

[bib33] Liddell B.J., Brown K.J., Kemp A.H., Barton M.J., Das P., Peduto A., Gordon E., Williams L.M. (2005). A direct brainstem-amygdala-cortical ‘alarm’ system for subliminal signals of fear. Neuroimage.

[bib34] Löw A., Weymar M., Hamm A.O. (2015). When threat is near, get out of here: dynamics of defensive behavior during freezing and active avoidance. Psychol. Sci..

[bib35] Macedo C.E., Martinez R.C., Brandão M.L. (2006). Conditioned and unconditioned fear organized in the inferior colliculus are differentially sensitive to injections of muscimol into the basolateral nucleus of the amygdala. Behav. Neurosci..

[bib36] McNaughton N., Corr P.J. (2004). A two-dimensional neuropsychology of defense: fear/anxiety and defensive distance. Neurosci. Biobehav. Rev..

[bib37] Mechias M.L., Etkin A., Kalisch R. (2010). A meta-analysis of instructed fear studies: implications for conscious appraisal of threat. Neuroimage.

[bib38] Mobbs D., Petrovic P., Marchant J.L., Hassabis D., Weiskopf N., Seymour B., Dolan R.J., Frith C.D. (2007). When fear is near: threat imminence elicits prefrontal-periaqueductal gray shifts in humans. Science.

[bib39] Montoya E.R., van Honk J., Bos P.A., Terburg D. (2015). Dissociated neural effects of cortisol depending on threat escapability. Hum. Brain Mapp..

[bib40] Morgan B., Terburg D., Thornton H.B., Stein D.J., van Honk J. (2012). Paradoxical facilitation of working memory after basolateral amygdala damage. PLoS ONE.

[bib41] Moscarello J.M., LeDoux J.E. (2013). Active avoidance learning requires prefrontal suppression of amygdala-mediated defensive reactions. J. Neurosci..

[bib42] Namburi P., Beyeler A., Yorozu S., Calhoon G.G., Halbert S.A., Wichmann R., Holden S.S., Mertens K.L., Anahtar M., Felix-Ortiz A.C. (2015). A circuit mechanism for differentiating positive and negative associations. Nature.

[bib43] Paxinos G., Watson C. (1997). The Rat Brain in Stereotaxic Coordinates.

[bib44] Phelps E.A., LeDoux J.E. (2005). Contributions of the amygdala to emotion processing: from animal models to human behavior. Neuron.

[bib45] Phillips A.G., Ahn S., Howland J.G. (2003). Amygdalar control of the mesocorticolimbic dopamine system: parallel pathways to motivated behavior. Neurosci. Biobehav. Rev..

[bib46] Solano-Castiella E., Anwander A., Lohmann G., Weiss M., Docherty C., Geyer S., Reimer E., Friederici A.D., Turner R. (2010). Diffusion tensor imaging segments the human amygdala in vivo. Neuroimage.

[bib47] Terburg D., Morgan B.E., Montoya E.R., Hooge I.T., Thornton H.B., Hariri A.R., Panksepp J., Stein D.J., van Honk J. (2012). Hypervigilance for fear after basolateral amygdala damage in humans. Transl. Psychiatry.

[bib48] Tovote P., Esposito M.S., Botta P., Chaudun F., Fadok J.P., Markovic M., Wolff S.B.E., Ramakrishnan C., Fenno L., Deisseroth K. (2016). Midbrain circuits for defensive behaviour. Nature.

[bib49] Tye K.M., Prakash R., Kim S.Y., Fenno L.E., Grosenick L., Zarabi H., Thompson K.R., Gradinaru V., Ramakrishnan C., Deisseroth K. (2011). Amygdala circuitry mediating reversible and bidirectional control of anxiety. Nature.

[bib50] Tzourio-Mazoyer N., Landeau B., Papathanassiou D., Crivello F., Etard O., Delcroix N., Mazoyer B., Joliot M. (2002). Automated anatomical labeling of activations in SPM using a macroscopic anatomical parcellation of the MNI MRI single-subject brain. Neuroimage.

[bib51] van Honk J., Schutter D.J., d’Alfonso A.A., Kessels R.P., de Haan E.H. (2002). 1 hz rTMS over the right prefrontal cortex reduces vigilant attention to unmasked but not to masked fearful faces. Biol. Psychiatry.

[bib52] van Honk J., Peper J.S., Schutter D.J. (2005). Testosterone reduces unconscious fear but not consciously experienced anxiety: implications for the disorders of fear and anxiety. Biol. Psychiatry.

[bib53] van Honk J., Eisenegger C., Terburg D., Stein D.J., Morgan B. (2013). Generous economic investments after basolateral amygdala damage. Proc. Natl. Acad. Sci. USA.

[bib54] Van Hougenhouck-Tulleken W., Chan I., Hamada T., Thornton H., Jenkins T., McLean W.H., McGrath J.A., Ramsay M. (2004). Clinical and molecular characterization of lipoid proteinosis in Namaqualand, South Africa. Br. J. Dermatol..

[bib55] Viviani D., Charlet A., van den Burg E., Robinet C., Hurni N., Abatis M., Magara F., Stoop R. (2011). Oxytocin selectively gates fear responses through distinct outputs from the central amygdala. Science.

