# Supplementary material for: The Basolateral Amygdala Is Essential for Rapid Escape: A Human and Rodent Study
Source: Cell. 2018 Oct 18;175(3):723–735.e16. doi: 10.1016/j.cell.2018.09.028 (PMC6198024; doi:10.1016/j.cell.2018.09.028)
Supplement: Document S1. Tables S1–S3 [file mmc1.pdf]

Cell, Volume *175*

Supplemental Information

The Basolateral Amygdala Is Essential for Rapid Escape: A Human and Rodent Study

David Terburg, Diego Scheggia, Rodrigo Triana del Rio, Floris Klumpers, Alexandru Cristian Ciobanu, Barak Morgan, Estrella R. Montoya, Peter A. Bos, Gion Giobellina, Erwin H. van den Burg, Beatrice de Gelder, Dan J. Stein, Ron Stoop, and Jack van Honk

SUPPLEMENTAL TABLES

Table S1: Related to Figure 1. IQ and age of the UWDs and HC samples in both (ASR and fMRI) human experiments with standard deviations (*SD*) and *p*-value for the independent samples *t*-test of the group differences.

	UWDs						HCs		
	**UWD1**	**UWD2**	**UWD3**	**UWD4**	**UWD6**	**mean (*SD*)**	***N***	**mean (*SD*)**	***p*-value**
**Age ASR**	24	31	35	49	37	35.4 (9.1)	14	32.4 (6.6)	0.44
**Age fMRI**	27	34	38	52	40	38.4 (9.1)	15	34.7 (5.7)	0.30
**IQ ASR**	98	84	87	81	83	86.4 (6.3)	14	86.6 (3.4)	0.93
**IQ fMRI**							15	86.1 (4.8)	0.90

Table S2: Related to Figure 2. Threat potentiated startle for each distance condition and both experimental groups. Values represent estimated marginal mean differences from the overall GEE model. ***p*<0.01, ****p*<0.001. See also Fig. 3.

Group	Distance	Threat potentiated startle	95% Confidence interval
**UWDs**	**Distant**	0.13	-0.11 to 0.38
	**Imminent**	0.57***	0.32 to 0.82
	**Inescapable**	0.75***	0.49 to 1.00
**HCs**	**Distant**	0.01	-0.13 to 0.16
	**Imminent**	0.22**	0.06 to 0.37
	**Inescapable**	0.49***	0.33 to 0.64

Table S3: Related to Figure 3. Peak-voxel MNI-coordinates and statistics from the significant clusters in the TET. Significant clusters are identified whole-brain or within the respective region of interest (indicated with *) using a *p*<0.005 voxel-detection threshold followed by a *p*<0.05 FWE cluster correction.

Structure	Hemisphere	Cluster size	*p*-value	Peak *T*-value	MNI-coordinate X	Y	Z
			**Threat>Safe**				
Anterior Insula	Left	1039	<0.001	4.58	-50	16	0
	Right	1538	<0.001	5.41	36	22	6
Inferior Frontal Gyrus	Right	s.c.		4.31	32	54	-6
Anterior Cingulate Cortex / Supplemental Motor Area	Both	2912	<0.001	5.33	8	24	38
Motor Cortex	Right	s.c.		5.16	36	0	58
Striatum / Thalamus	Both	770	0.001	4.51	10	0	2
Medial Temporal Cortex	Right	292	0.044	4.23	62	-44	24
Periaqueductal Gray*	Both	71	0.018	3.72	4	-30	-4
			**Inescapable: Threat>Safe**				
Occipital Cortex	Left	1643	0.001	5.02	-14	-56	0
	Right	786	0.03	3.64	18	-48	-6
Anterior Insula	Left	877	0.019	4.11	-48	18	-2
	Right	790	0.03	4.09	40	24	4
Anterior Cingulate Cortex / Supplemental Motor Area	Both	1050	0.008	4.02	8	24	38
Periaqueductal Gray*	Both	36	0.028	3.15	2	-28	-2
			**Imminent: Threat>Safe**				
Motor Cortex	Right	899	0.017	4.72	36	0	58
Inferior Frontal Gyrus	Right	2129	<0.001	4.31	40	46	12
Anterior Insula	Right	s.c.		4.05	36	24	-2
			**Distant: Threat>Safe**				
Anterior Cingulate Cortex / Supplemental Motor Area	Both	2206	<0.001	4.78	16	18	42
Motor Cortex	Left	s.c.		4.29	-28	2	52
	Right	s.c.		4.13	38	-2	52
Thalamus	Both	1386	0.002	4.4	-10	-4	-8
Striatum	Both	s.c.		4.03	-18	2	12
Medial Temporal Cortex	Left	1400	0.002	4.12	-48	-20	26
Anterior Insula	Left	1057	0.008	3.79	-38	18	-6
			**Group x Threat x Distance**				
Pons*	Both	229	0.023	9.92#	6	-26	-32
			**UWD: Threat x Distance**				
Pons*	Both	162	0.011	10.94#	4	-28	-32
			**UWD: Threat>Safe (Inescapable>Distant)**				
Pons*	Both	209	0.029	3.95	4	-26	-34
			**UWD: Threat>Safe (Imminent>Distant)**				
Pons*	Both	191	0.033	4.01	4	-30	-32
			**PPI: HC>UWD**				
Central / Medial Amygdala*	Right	16	0.027	3.51	22	-10	-6

Statistics are FWE cluster-corrected at *p*<0.05 with cluster defining threshold *p*<0.005
F-test with FWE corrected voxel threshold *p*<0.05
s.c. same cluster
